# Targeted mRNA delivery with bispecific antibodies that tether LNPs to cell surface markers

**DOI:** 10.1016/j.omtn.2025.102520

**Published:** 2025-03-19

**Authors:** Bettina Dietmair, James Humphries, Timothy R. Mercer, Kristofer J. Thurecht, Christopher B. Howard, Seth W. Cheetham

**Affiliations:** 1 Australian Institute for Bioengineering and Nanotechnology, The University of Queensland, Brisbane, QLD, Australia; 2 BASE Facility, The University of Queensland, Brisbane, QLD, Australia; 3 Centre for Advanced Imaging, ARC Research Hub for Advanced Manufacture of Targeted Radiopharmaceuticals, The University of Queensland, St Lucia, QLD 4072, Australia

**Keywords:** MT: Delivery Strategies, mRNA, LNP, drug delivery, targeted delivery, cancer, bispecific antibodies, lipid nanoparticles

## Abstract

Efficient delivery of mRNA-lipid nanoparticles (LNPs) to specific cell types remains a major challenge for mRNA therapeutics. Conventional targeting approaches involve modifying the lipid composition or functionalizing the surface of LNPs, which complicates manufacturing and alters nanoparticle size, charge, and stealth, impacting their delivery and immunogenicity. Here, we present a generalizable method for targeted mRNA-LNP delivery that uses bispecific antibodies (BsAbs) to form a bridge between LNPs and cell surface markers. BsAbs can be combined with LNPs or administered first, binding to surface proteins on target cells and later retaining unmodified LNPs in affected tissues. We demonstrate the efficient and cell-type-specific delivery of mRNA-LNPs beyond the liver, targeting epidermal growth factor receptor (EGFR)- and folate hydrolase 1 (PSMA)-positive cells *in vitro* and *in vivo*. The flexibility of this technology, achieved by substituting the cell-targeting region of the BsAbs, enables the rapid development of next-generation targeted mRNA drugs.

## Graphical abstract

**Figure:**
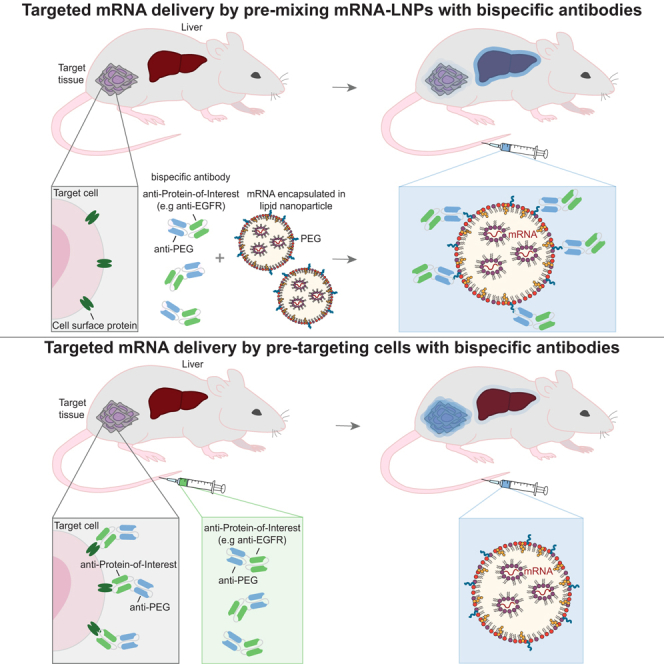

## Teaser

Most tissues are inaccessible for systemic administration of lipid nanoparticle-formulated mRNA therapeutics. Customizable bispecific antibodies bind to lipid nanoparticles and proteins on target cells, enabling cell-specific mRNA delivery and broadening the scope of mRNA applications.

## Introduction

mRNA therapies are rapidly emerging as a new class of drugs with the potential to treat a wide range of human diseases. Beyond vaccines, mRNA drugs in development include treatments for cancers,^1^^,^^2^ autoimmunity,^3^ and hereditary diseases.^4^ The efficacy of mRNA drugs depends on the ability to deliver mRNA efficiently to specific cell types. Polyethylene glycol (PEG)-coated lipid nanoparticles (LNPs) are clinically validated as safe and effective mRNA delivery systems.^5^^,^^6^^,^^7^ However, following intravenous administration, LNP accumulation and mRNA expression occur mainly in the liver.^8^^,^^9^ The liver tropism of LNPs limits the potential of mRNA for treating extrahepatic diseases.

Current attempts to target mRNA-LNPs *in vivo* employ high drug doses and remain restricted to the liver, lungs, and spleen.^10^^,^^11^ We report a customizable solution to achieve cell-specific mRNA delivery to previously inaccessible tissues using bispecific antibodies (BsAbs). The BsAbs comprise two linked single-chain variable fragments (scFvs) that can bind to PEG on the exterior of LNPs and to a protein enriched on the target cell surface. BsAbs can be attached to the surface of LNPs before administration (pre-mixing). We further present pre-targeting, a novel approach for targeted mRNA-LNP delivery, whereby cells are initially exposed to BsAbs, followed by administration of unmodified LNPs.^12^ Targeting with BsAbs enabled efficient mRNA drug delivery beyond the liver. Sequential administration of BsAbs and mRNA-LNPs demonstrated specificity for epidermal growth factor receptor (EGFR) and folate hydrolase 1 (PSMA) *in vitro* and *in vivo*, significantly improving mRNA-LNP delivery to the target tissue and reducing accumulation in off-target organs. The new concept of pre-targeting can be adapted to different cell surface antigens to achieve safe and efficacious targeted mRNA delivery, enabling the development of mRNA medicines for broader applications.

## Results

### Pre-targeting with BsAbs improves cell-specific mRNA-LNP delivery

**Figure 1:**
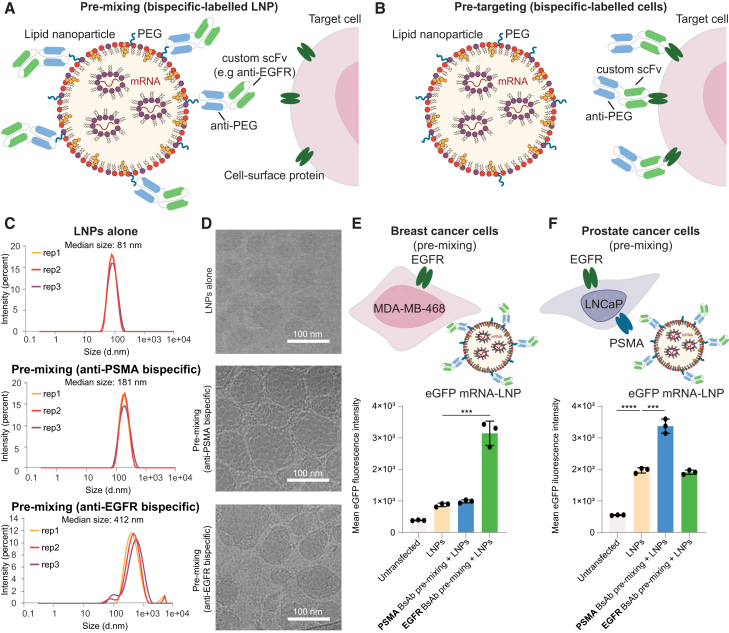
Pre-mixing with bispecific antibodies alters physicochemical properties and delivery of mRNA-LNPs (A) During pre-mixing, bispecific antibodies (BsAbs) bind to polyethylene glycol (PEG) on the surface of mRNA-loaded lipid nanoparticles (LNPs). The second binding region of the BsAb can bind to the surface protein on the target cell. (B) For pre-targeting, cells are exposed to BsAbs that can specifically bind to surface proteins. mRNA-carrying LNPs can then bind to the PEG-specific binding region of the BsAb. (C) Triplicate dynamic light scattering measurements of size distribution of EGFP-mRNA LNPs without BsAbs, after pre-mixing with PSMA-PEG BsAbs, and after pre-mixing with EGFR-PEG BsAbs. (D) Cryogenic transmission electron microscopy images of EGFP-mRNA LNPs without BsAbs, after pre-mixing with PSMA-PEG BsAbs, and after pre-mixing with EGFR-PEG BsAbs. Scale bar: 100 nm. (E and F) Mean EGFP fluorescence intensity of cells transfected with untargeted EGFP-mRNA LNPs, with LNPs pre-mixed with PSMA-PEG BsAbs, or LNPs pre-mixed with EGFR-PEG BsAbs, respectively, for (E) MDA-MB-468 breast cancer cells (PSMA−ve and EGFR+ve) and (F) LNCaP prostate cancer cells (PSMA+ve and EGFR+ve). Mean EGFP fluorescence intensity was measured using flow cytometry. Statistical analysis was performed using two-tailed t tests assuming equal variance. Bars represent the mean value, and error bars indicate standard deviation (*n* = 3). ∗∗∗*p* < 0.001 and ∗∗∗∗*p* < 0.0001.

To determine if cell surface antigen-specific BsAbs enable targeted mRNA-LNP delivery, we synthesized and encapsulated enhanced green fluorescent protein (EGFP) mRNA in LNPs (Figure S1A). As active targeting approaches commonly attach the targeting agent to the LNPs (pre-mixing; Figure 1A), not to the target cells (pre-targeting; Figure 1B), we first characterized the effect of surface functionalization on LNP properties. Pre-mixing of the mRNA-LNPs with BsAbs increased the median size of the mRNA-LNP from 82 to 181 and 412 nm for anti-PSMA and anti-EGFR BsAbs, respectively (Figure 1C). LNP size and polydispersity doubled after 5 min of incubation with anti-EGFR BsAbs and tripled by 40 min (Figure S1B). BsAb coating altered LNP morphology, reduced particle uniformity, and induced aggregation (Figures 1C and 1D), which may contribute to LNP behavior *in vivo.*^13^^,^^14^ For intravenous administration and tissue penetration, nanoparticle sizes below 150 nm are preferred.^15^

To evaluate cell-type-specific delivery of mRNA-LNPs pre-mixed with BsAbs, we used MDA-MB-468 breast cancer cells, which express EGFR but not PSMA, and LNCaP prostate cancer cells, which express both EGFR and PSMA (Figure S1C). Both cell lines express the low-density lipoprotein receptor (LDLR), and cell culture medium was supplemented with serum containing apolipoprotein,^16^ as the uptake of untargeted LNPs *in vivo* is largely dependent on apolipoprotein E adsorption to the nanoparticle surface and subsequent internalization via the LDLR on hepatocytes.^17^ Pre-mixing of LNPs with anti-PEG:anti-EGFR BsAbs enhanced mRNA-LNP delivery to MDA-MB-468 (Figure 1E). LNP surface functionalization with anti-PEG:anti-PSMA BsAbs did not improve mRNA-LNP delivery to the PSMA-negative cell line. To confirm the cell-type specificity of BsAb-mediated targeting, we analyzed mRNA-LNP delivery to EGFR+ve (EGFR-positive), PSMA+ve (PSMA-positive) LNCaP prostate cancer cells (Figure 1F). EGFP mRNA-LNP delivery significantly improved when mRNA-LNPs were pre-mixed with anti-PEG:anti-PSMA BsAbs. Pre-mixing with anti-PEG:anti-EGFR BsAbs had no effect on EGFP expression compared to untargeted LNPs.

**Figure 2:**
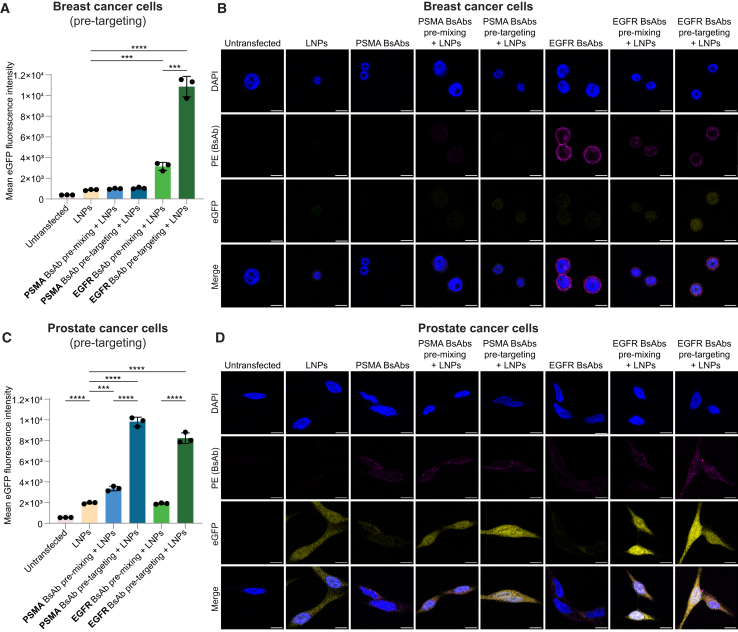
Pre-targeting of cells with bispecific antibodies improves cell-specific delivery of mRNA-LNPs (A) Mean EGFP fluorescence intensity of MDA-MB-468 breast cancer cells (PSMA−ve and EGFR+ve) transfected with EGFP-mRNA LNPs. PSMA-PEG BsAbs or EGFR-PEG BsAbs were pre-mixed with LNPs or pre-targeted to MDA-MB-468 cells, respectively. (B) Confocal microscopy images of MDA-MB-468 after addition of EGFP mRNA-LNPs, PSMA-PEG, or EGFR-PEG BsAbs, BsAbs pre-mixed with LNPs, or pre-targeting with BsAbs followed by addition of LNPs. (C) Mean EGFP fluorescence intensity of LNCaP prostate cancer cells (PSMA+ve and EGFR+ve) transfected with EGFP-mRNA LNPs. PSMA-PEG BsAbs or EGFR-PEG BsAbs were pre-mixed with LNPs or pre-targeted to LNCaP cells, respectively. (D) Confocal microscopy images of LNCaP after addition of EGFP mRNA-LNPs, PSMA-PEG, or EGFR-PEG BsAbs, BsAbs pre-mixed with LNPs, or pre-targeting with BsAbs followed by addition of LNPs. Confocal microscopy images were taken at 63× magnification and show EGFP expression (yellow), BsAb localization (protein L-phycoerythrin conjugate labeled; magenta), and 4′,6-diamidino-2-phenylindole DNA stain (DAPI; blue) 4 h after addition of LNPs. Scale bar: 10 μm. Mean EGFP fluorescence intensity was measured using flow cytometry. Statistical analysis was performed using two-tailed t tests assuming equal variance. Bars represent the mean value, and error bars indicate standard deviation (*n* = 3). ∗∗∗*p* < 0.001 and ∗∗∗∗*p* < 0.0001.

We then compared the established pre-mixing protocol to the pre-targeting of cells with BsAbs. Pre-targeting MDA-MB-468 breast cancer cells with anti-PEG:anti-EGFR BsAbs significantly increased EGFP expression compared to pre-mixing (12- vs. 3-fold improvement over mRNA-LNPs alone; Figures 2A, 2B, and S1D). The duration of pre-mixing had no impact on mRNA-LNP delivery (Figure S1E). Similarly, pre-targeting LNCaP prostate cancer cells reached almost 3-fold improvement with PSMA BsAbs and 4-fold improvement with EGFR BsAbs compared to pre-mixing (Figures 2C, 2D, and S1F). Compared to untargeted LNP transfection, pre-targeting with EGFR-PEG and PSMA-PEG achieved 4- and 5-fold EGFP expression, respectively. Notably, pre-targeting with EGFR-PEG BsAbs enhanced mRNA-LNP delivery to EGFR+ve LNCaP cells, while pre-mixing did not improve uptake.

In summary, pre-mixing and pre-targeting with anti-PEG BsAbs facilitate cell-type-specific mRNA-LNP delivery *in vitro*. The pre-targeting approach is applicable to different cell lines and target antigens and is more efficient than pre-mixing. Active targeting employs interactions with a cell surface receptor to promote specificity and internalization via endocytosis.^18^ As BsAbs are below the size range in which receptor-mediated endocytosis is triggered,^19^^,^^20^ they accumulate on the plasma membrane and are internalized once mRNA-LNPs bind (Figures 2B and 2D). In addition to maintaining the physicochemical properties of mRNA-LNPs by pre-targeting, avidity might improve delivery with more BsAb binding sites available on cell surfaces during pre-targeting than PEG on LNPs during pre-mixing. Increased avidity could explain improved mRNA-LNP delivery to LNCaP cells when pre-targeting with EGFR-PEG BsAbs despite lower EGFR expression on the cell surface (Figure S1C). As pre-targeting MDA-MB-468 with EGFR-PEG BsAbs achieved the greatest improvement over untargeted and pre-mixed LNP delivery *in vitro*, we tested this condition *in vivo*.

### Pre-targeting with BsAbs improves targeted mRNA-LNP delivery *in vivo*

**Figure 3:**
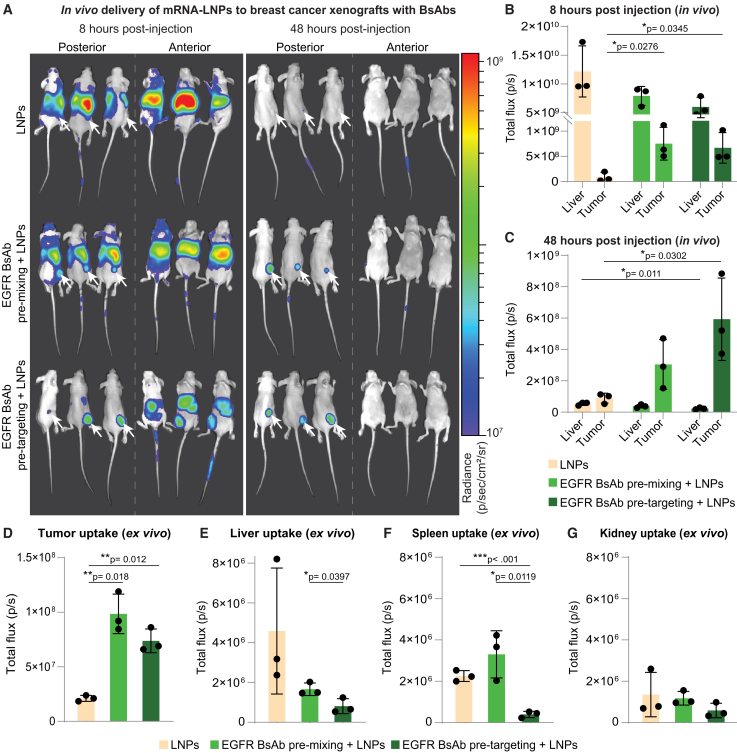
Pre-targeting with bispecific antibodies improves targeted delivery of mRNA-LNPs *in vivo* (A) *In vivo* bioluminescence images of MDA-MB-468 tumor-bearing mice injected with untargeted luciferase mRNA-LNPs, LNPs pre-mixed with EGFR-PEG BsAbs, or mice pre-injected with EGFR-PEG BsAbs followed by administration of mRNA-LNPs. White arrows indicate posterior tumor localization. (B and C) *In vivo* bioluminescence in the liver compared to tumor for different targeting approaches (B) 8 and (C) 48 h after luciferase mRNA-LNP administration. (D–G) *Ex vivo* bioluminescence imaging of (D) tumor, (E) liver, (F) spleen, and (G) kidney tissue at 48 h post-injection. Background was subtracted based on a saline-injected mouse. Statistical analysis was performed using two-tailed t tests assuming equal variance. Bars represent the mean value, and error bars indicate standard deviation (*n* = 3).

To evaluate the efficacy of BsAb-targeted mRNA-LNP delivery *in vivo*, we synthesized and encapsulated firefly luciferase mRNA in LNPs (Figures S2A–S2C) and pre-mixed or pre-targeted with EGFR-PEG BsAbs for intravenous administration to BALB/c nude mice with subcutaneous MDA-MB-468 xenografts (Figure 3A). Pre-mixing and pre-targeting with EGFR-PEG BsAbs significantly increased mRNA-LNP delivery to the tumor tissue (over 8- and 7-fold) while reducing the radiance in the liver by a third and half compared to untargeted LNPs, respectively, 8 h after luciferase mRNA-LNP administration (Figures 3B and S2D). After 48 h, luciferase expression in the liver was reduced across all delivery groups (Figures 3C and S2E). In contrast, luminescence in the tumor remained consistent with the 8-h time point in pre-targeted animals, maintaining significant levels over untargeted LNPs and only decreasing by around 10%. Radiance in the pre-mixing group tumors was reduced by 60% compared to the measurement after 8 h.

*Ex vivo* analysis of tumor signal confirmed *in vivo* biodistribution results, showing comparable luminescence between pre-mixing and pre-targeting cohorts with more than 4.6- and 3.5-fold elevated radiance compared to untargeted mRNA-LNP administration, respectively (Figures 3D and S2F). Consistently, total flux in the liver was highest in the untargeted LNP group and significantly lower in mice treated with pre-targeting compared to pre-mixing of BsAbs and LNPs (Figures 3E and S2G). Likewise, spleen uptake was the lowest in pre-targeted mice compared to over 5- and 8-fold increased spleen signals for the administration of untargeted and pre-mixed mRNA-LNPs, respectively (Figure 3F). The high splenic uptake of LNPs pre-mixed with BsAbs could result from changes in physicochemical properties, including a large protein corona around the LNPs^14^^,^^21^ and a charge shift from positively charged, unmodified LNPs to negatively charged, BsAb pre-mixed LNPs^22^ (Figure S2H). Lower spleen accumulation of unmodified mRNA LNPs in the pre-targeting group, compared to untargeted LNPs, could be a result of higher tumor uptake, leaving less nanoparticles in circulation for off-target accumulation. Radiance was low in the heart, blood, and the second major clearance organ, kidney (Figures 3G and S2G).

Confirming the *in vitro* results, targeting mRNA-LNPs with EGFR-PEG BsAbs *in vivo* resulted in mRNA delivery to EGFR+ve tumors and reduced delivery to the liver, demonstrating specificity and efficacy compared to delivery of untargeted LNPs. Pre-targeting led to sustained mRNA expression in the tumor and the lowest signals in the liver and spleen compared to pre-mixing of LNPs with BsAbs or administration of untargeted LNPs.

## Discussion

We demonstrated efficient mRNA-LNP targeting *in vitro* and *in vivo* using BsAbs specific to LNPs and cell surface markers. Temporal separation of BsAb and nanoparticle administration via pre-targeting achieved superior mRNA uptake and expression. This is the first time pre-targeting has been characterized for mRNA-LNPs. One explanation for the improved biodistribution of mRNA-LNPs during pre-targeting, relative to pre-mixed systems, could be reduced protein fouling on the particle surface.^12^ Reduced protein adsorption to the LNP surface can decrease receptor-mediated hepatic uptake^17^ and immune recognition,^23^ thus extending the circulation time and enabling the nanoparticles to reach peripheral target tissues. Maintained particle properties, including size and charge, can facilitate LNP uptake into target cells. Pre-targeting further conserves ligand affinity *in vivo*, which can vary in conjugated antibody systems due to reaction conditions and the attachment of antibodies in an unfavorable orientation.^24^ BsAbs enable mRNA delivery to previously inaccessible tissues, which broadens the scope of conditions addressable with mRNA therapeutics. Lower mRNA dosage and reduced uptake in off-target organs, such as the liver and spleen, could reduce the toxicity of mRNA therapies. While mRNA-LNPs were cleared from the metabolically active liver,^23^ efficient mRNA expression persisted exclusively in the tumor. The structure of the BsAbs facilitates substitution of the antigen-specific binding domain,^25^^,^^26^^,^^27^ enabling customized targeting of unmodified, PEG-containing mRNA carriers to markers present on individual patient cell types. The separate production of the mRNA-LNP and targeting agent (BsAb) greatly streamlines manufacture and quality control compared to functionalized mRNA-LNPs.^28^ Broad applicability to PEGylated nanocarriers and decreased complexity of production and purification enhance the scalability of pre-targeting.^29^ The well-established clinical use and safety of mRNA-LNPs^30^ and BsAbs^31^ provide strong precedence for a streamlined clinical translation of this technology. While sequential drug administration is common in cancer therapy,^32^ this treatment regimen should be considered for clinical practice in other applications. Efficient and adaptable targeting of mRNA-LNPs enables the rapid development of next-generation mRNA drugs, including protein replacement therapies and gene editing applications for incurable diseases.

## Materials and methods

Detailed methods can be found in the supplemental materials and methods.

## Data availability

Data supporting the findings of this study are available within the article and its supplemental information.

## Acknowledgments

We acknowledge the following sources of funding and support: the Australian Government Research Training Program (RTP) Scholarship to B.D., Innovation Connections funding to S.W.C. and T.R.M., the 10.13039/501100000925National Health and Medical Research Council (GNT2019056 to K.J.T. and GNT2014002 and GNT1161832 to T.R.M.), the 10.13039/501100000923Australian Research Council (IH220100017 to K.J.T. and DE230100036 to S.W.C.), the Medical Research Future Fund (MRFCRI000063 and MRFNCRI000089 to S.W.C. and T.R.M.), the National Collaborative Research Infrastructure Strategy (NCRIS) to T.R.M. and S.W.C., 10.13039/501100020111Therapeutic Innovation Australia (TIA) to T.R.M. and S.W.C., Tour de Cure to S.W.C., and 10.13039/501100001794The University of Queensland to S.W.C. and T.R.M. The authors acknowledge the facilities and the scientific and technical assistance of the Australian National Fabrication Facility (ANFF, Queensland Node), the Centre for Microscopy and Microanalysis (CMM), the National Imaging Facility (NIF), and BASE at The University of Queensland. BASE is supported by TIA. TIA is supported by the Australian government through the National Collaborative Research Infrastructure Strategy (NCRIS) program.

## Author contributions

B.D. and J.H. performed the experiments and the analysis. C.B.H. and S.W.C. conceived the project. S.W.C., C.B.H., T.R.M., and K.J.T. funded the study. All authors contributed to writing the paper.

## Declaration of interests

T.R.M. and S.W.C. have received research funding from Oxford Nanopore Technologies, Sartorius Stedim Australia, and Sanofi. T.R.M. and S.W.C. have received support for conference attendance, travel, and accommodations from Moderna and Oxford Nanopore Technologies.

## Supplemental information

Document S1. Figures S1 and S2, Table S1, and Supplemental materials and methods

Document S2. Article plus supplemental information
