# Supplementary material for: Targeted mRNA delivery with bispecific antibodies that tether LNPs to cell surface markers
Source: Mol Ther Nucleic Acids. 2025 Mar 19;36(2):102520. doi: 10.1016/j.omtn.2025.102520 (PMC11999258; doi:10.1016/j.omtn.2025.102520)
Supplement: Document S1. Figures S1 and S2, Table S1, and Supplemental materials and methods [file mmc1.pdf]

OMTN, Volume *36*

Supplemental information

Targeted mRNA delivery with bispecific antibodies that tether LNPs to cell surface markers

Bettina Dietmair, James Humphries, Timothy R. Mercer, Kristofer J. Thurecht, Christopher B. Howard, and Seth W. Cheetham

Supplemental materials and methods

Template production

eGFP and firefly luciferase mRNA templates were designed with the CleanCap® AG promoter,[1] human alpha-globin 5′ UTR and the mouse alpha-globin 3′ UTR sequences and synthesized as gBlocks™ HiFi Gene Fragments (IDT™). 0.5 ng of Gene Fragments, forward primer (10 µM), reverse primer (10 µM), and NEB® Q5® HotStart 2 × master mix were combined at room temperature in a 100 µL PCR reaction and placed on ice. The template was amplified using the thermocycler program described in **Table S1**. Eight PCR reactions were purified using the Qiagen® QIAquick® PCR cleanup kit, according to the manufacturer's instructions. The PCR product was eluted in 30 µL of ultrapure water, analyzed by gel electrophoresis and quantified using UV spectrophotometry.

mRNA production

Amplified Gene Fragments were used as template for mRNA *in vitro* transcription (IVT) at a concentration of 50 µg/mL. IVT was performed using 16 µg/mL T7 RNA polymerase (New England Biolabs; NEB® M0251), ribonucleotides (6 mM ATP, 5 mM CTP, 5 mM GTP; NEB®), 5 mM N1-methylpseudouridine-5'-triphosphate (TriLink® BioTechnologies, TRN1081), 4 mM CleanCap® AG reagent (TriLink® BioTechnologies, TRN7113), transcription buffer (40 mM Tris·HCl pH 8.0, 16.5 mM magnesium acetate, 10 mM dithiothreitol (DTT), 20 mM spermidine, 0.002 % (v/v) Triton X-100), 2 U/mL yeast inorganic pyrophosphatase (NEB®) and 1000 U/mL murine RNase inhibitor (NEB®). The IVT reaction was incubated at 37 °C for three hours and terminated by incubation with 200 U/mL NEB® DNase I at 37 °C for 15 minutes. The mRNA product was purified using a Monarch® RNA Cleanup Kit (NEB®) according to the manufacturer's instructions, eluted in 1 mM sodium citrate and sterile filtered with a 0.22 µm syringe filter. The mRNA was quantified by UV spectrophotometry and integrity confirmed using the Agilent® TapeStation™.

LNP production

For LNP formulation, a total lipid concentration of 15 mg/mL was used in the molar ratio of 50 SM-102 : 10 DSPC : 38.5 cholesterol : 1.5 DMG-PEG2000. The lipid mixture was made up to 3.75 mL with molecular grade 100 % ethanol. 1.5 mg of purified mRNA were combined with 11.25 mL of 0.1 M sodium acetate, pH 4.0. Formulation was performed on the NanoAssemblr® Ignite™ platform with the following parameters: total volume 13.5 mL, total flow rate 12 mL/min and flow rate ratio (aqueous:organic) 3:1. The mRNA-LNPs were dialyzed using the Slide-A-Lyzer™ dialysis cassette

(10K MWCO) and concentrated using an Amicon® Ultra-15 Centrifugal Filter Unit (10 kDa MWCO). The concentrated mRNA-LNPs were filtered with a 0.22 µm syringe filter and 0.2 volumes of 50 % sucrose were added by gentle pipetting for a final 10 % concentration. Encapsulation efficiency was measured on an Infinite® 200 PRO microplate reader (Tecan®) using the Quant-iT™ RiboGreen® RNA Reagent Kit (Life Technologies™). Determined encapsulation efficiencies for eGFP and luciferase mRNA-LNPs were 98.0 % and 98.8 %, respectively.

Bispecific antibody production

Bispecific antibodies were produced as previously described.[2] An scFv specific for PEG was linked to an scFv specific for human epidermal growth factor receptor[3] or folate hydrolase 1[4] via a glycine serine linker (G4S). The BsAb sequences were codon optimized for expression in *C. griseus* cells, included a κ light chain leader sequence for protein secretion, a 6 × Histidine motif at the N-terminus of the BsAb and a c-myc epitope tag at the C-terminus for purification and detection of the BsAb. The BsAb genes were cloned into the pcDNA™ 3.1 (+) mammalian expression plasmid (Invitrogen™) using HindIII and NotI restriction sites. For transient transfection, the plasmid DNA was transfected into ExpiCHO™ cells (Gibco™) using 2 µg DNA per mL cells at a concentration of 6×10^6 mL^{-1} cells. For a 200 mL cell volume transfection, 200 µg DNA in 8 mL of OptiPRO™ serum free medium (SFM; Gibco™) were mixed with 7.4 mL OptiPRO™ SFM containing 640 µL ExpiFectamine™ (Gibco™) for five minutes prior to transfecting ExpiCHO™ cells. The transfected cells were cultured in ExpiCHO™ expression medium (Gibco™) at 37 °C, 7.5% CO_2, 70 % humidity, with shaking at 130 rpm for 24 h, before feeding with 10 % ExpiCHO™ Feed (Gibco™) and 1.2 mL ExpiFectamine™ enhancer reagent (Gibco™) and returning cultures to 32 °C, 7.5 % CO_2, 70 % humidity, with shaking at 130 rpm.

Following transfection, the cells were pelleted by centrifugation at 5250 × g for 30 minutes and the supernatant was collected and filtered through a 0.22 µm PES membrane (Sartorius®). The BsAbs were purified from the supernatant using a 5 mL HisTrap™ excel column (Cytiva™), eluting the protein with 20×10^{-3} M sodium phosphate, 500×10^{-3} M sodium chloride, and 500×10^{-3} M Imidazole pH 7.4. BsAbs were then buffer exchanged into 1 × phosphate-buffered saline (PBS) using a HiPrep™ 26/10 column (Cytiva™). The final product was sterile filtered using a 0.2 µm polyethersulfone (PES) membrane filter (Sartorius®).

Characterisation of LNPs

LNP size and charge were measured on a Zetasizer® Ultra (Malvern Panalytical®). mRNA-LNPs were diluted 50-fold in distilled water (Invitrogen™). For pre-mixing, 10 × excess BsAbs (w/w) were added and samples were incubated for 60 minutes at room temperature. Ten-fold BsAb excess was used as lower ratios did not achieve maximum efficacy and higher ratios reduced delivery using the pre-mixing method (**Figure S2I**).

Cell culture

MDA-MB-468 human breast cancer cells were cultured in Dulbecco's modified Eagle's medium (DMEM; Gibco™), supplemented with 10 % (v/v) fetal bovine serum (FBS; Gibco™) and 1 × penicillin-streptomycin (P/S; 100 U/mL penicillin and 100 µg/mL streptomycin; Gibco™). LNCaP human prostate cancer cells were maintained in Roswell Park Memorial Institute medium (RPMI-1640; Sigma-Aldrich®), 10 % FBS and 1 × P/S. Cells were incubated at 37 °C in 5 % CO_2 and propagated for no more than 30 passages.

***In vitro* BsAb-targeted mRNA-LNP delivery**

For 70 % confluency at transfection, 1.9×10^5 MDA-MB-468 or LNCaP cells were plated into the wells of 24-well cell culture plates in 500 µL of appropriate cell culture medium. To enhance LNCaP adhesion, 24-well plates were coated with poly-D-lysine (Gibco™) according to manufacturer specifications. The cells were allowed to attach overnight before treatment. All treatments were incubated with cells for 60 minutes at 37 °C with 5 % CO_2. Treatments were diluted in Dulbecco's Phosphate-Buffered Saline (DPBS; Gibco™) and added in volumes of 10 µL. mRNA concentration per well was 60 ng and BsAb concentration was 600 ng. For washing steps, cells were rinsed twice with DPBS and fresh complete medium was added. Four hours after addition of LNPs, cells were prepared for flow cytometry.

For pre-mixed samples, eGFP mRNA-LNPs were incubated with BsAbs for 60 minutes at room temperature. The treatment was then added to the wells, incubated and washed. For pre-targeting, cells were incubated with BsAbs for 60 minutes, washed, incubated with eGFP mRNA-LNPs and washed again. The untargeted LNP samples were incubated with DPBS, washed, incubated with eGFP mRNA-LNPs and washed again.

Flow cytometry

To prepare samples for flow cytometry, medium was removed, cells were washed with DPBS, detached with 0.25 % 1 × trypsin (Gibco™), centrifuged at 200 × g for 5 minutes and resuspended in 250 µL flow buffer (DPBS, 2 % FBS, 2 mM ethylenediaminetetraacetic acid; EDTA). 7-aminoactinomycin D (7-AAD; Invitrogen™) viability stain was used and samples were incubated in the dark on ice for 30 minutes. 20,000 single cell events were recorded on a CytoFLEX™ Flow Cytometer (Beckman Coulter®) at a flow rate of 10 µL/s. eGFP and 7-AAD were excited using a 488 nm laser and emission was detected with a 525/40 bandpass and a 690/50 bandpass filter, respectively. Compensation and data analysis were performed using FlowJo™ v10.10.0. Software[5] (BD Life Sciences™). GraphPad Prism™ v10.1.2[6] (GraphPad Software) was used for statistical analysis and graphing. Statistical analysis was performed using two-tailed t-tests assuming equal variance with *$p < 0.05$, **$p < 0.01$, ***$p < 0.001$ and ****$p < 0.0001$.

Confocal microscopy

4 × 10^4 MDA-MB-468 cells or 6 × 10^4 LNCaP cells were seeded on coverslips in 24-well plates and incubated overnight at 37 °C with 5 % CO_2. For LNCaP cells, coverslips were coated with poly-D-lysine (Gibco™) according to manufacturer instructions. The cells were treated as described for *in vitro* BsAb-targeted mRNA-LNP delivery. To prepare the samples for confocal microscopy, the cells were fixed with 4 % paraformaldehyde (Novachem™) for 15 minutes at room temperature, washed three times with 1 × PBS (Gibco™), permeabilized and blocked with PBS buffer containing 0.1 % Triton X-100 (Sigma-Aldrich®) and 1 × bovine serum albumin (Sigma-Aldrich®) for one hour at room temperature. DAPI (BioLegend®) and Protein L-phycoerythrin conjugate staining (1:100; Cell Signaling Technology®) was performed for one hour at room temperature. The cells were washed three times with 1 × PBS and one time with Milli-Q® water before mounting on glass slides. Imaging was performed on a Zeiss® LSM® 710 inverted laser scanning confocal microscope at the Queensland node of the NCRIS-enabled Australian National Fabrication Facility (ANFF). Images were taken using a 63 × oil immersion objective for sequential scanning with excitation of DAPI, eGFP and phycoerythrin at 405 nm, 488 nm and 561 nm, respectively. FIJI[7] was used for image processing.

Cryo electron microscopy

Cryo-transmission electron microscopy was performed by the Centre for Microscopy and Microanalysis, UQ. Lacey carbon films on 300 mesh copper grids were used as sample carriers (Electron Microscopy

Sciences®) and glow discharged for 60 seconds at 10 % power using a Femto system for sample cleaning directly before usage (Diener Electronic®). The samples were cryo-fixed in liquid ethane at -184 ºC using a Leica EM-GP2 (Leica® Microsystems). 2.5 µL samples were blotted for three to four seconds, frozen at 20 ºC and 99 % relative humidity, and stored in liquid nitrogen until further processing. Image acquisition of the frozen hydrated LNPs was performed using a CryoARM™ 200 JEM-Z200 FSC (JEOL™) equipped with a K2™ Summit direct electron detector (Gatan®). The microscope was operated at 200 kV with the microscope energy filter set to a slit width of 20 eV. SerialEM[8,9] in combination with Gatan's Digital Micrograph was used for image acquisition. Samples were imaged at -180 ºC under low dose conditions. The exposure time for each record was 4 seconds with 40 subframes in counting mode. The dose rate on the detector was 8.5 e/px/s. Movie frames were motion corrected with the program's frame alignment feature and saved as a sum image of 40 subframes.[10,11]

***In vivo* imaging of mRNA-LNP biodistribution**

All studies were in accordance with guidelines of the Animal Ethics Committee of The University of Queensland (UQ; Approval 2023/AE000135), and the Australian Code for the Care and Use of Animals for Scientific Purposes. Female Balb/c nude mice (approximately 8 weeks of age) were acquired from the Ozgene-ARC® (Western Australia) and housed in temperature and humidity-controlled housing with *ad libitum* access to food and water.

Mice were subcutaneously injected with 5 × 10^6 MDA-MB-468 cells in the right flank (50 µL PBS, 27G needle). After tumors reached a palpable size (ca. 100-200 mm^3), the mice were segregated into three cohorts of n = 3 animals where the targeting strategy was varied. Mice in the untargeted LNP group were injected intravenously via the lateral tail vein with 10 µg of luciferase mRNA-LNPs diluted in 100 µL of PBS (29G needle). Mice in the pre-mixed cohort were injected intravenously with mRNA-LNPs pre-incubated with anti-PEG:anti-EGFR BsAbs at a ratio of 50 µg BsAbs per 10 µg mRNA 15 minutes prior to intravenous injection (diluted in 100 µL PBS, 29G needle). Mice in the pre-targeted cohort were injected intravenously with 1 mg of anti-PEG:anti-EGFR BsAbs (diluted in 100 µL of PBS with 300 mM NaCl) eight hours[12] prior to injection of 10 µg of mRNA-LNPs diluted in 100 µL of PBS (29G needle).

Mice were injected intraperitoneally with 150 mg kg^{-1} of D-Luciferin (VivoGlo™) 15 minutes prior to imaging using an IVIS® Lumina™ X5 imaging system (PerkinElmer®) eight hours and 48 hours post

injection of the LNPs. Data were acquired using the default acquisition settings, then post-processed to a binning factor of four. Segmented regions of interest (ROIs) were drawn to delineate the liver and tumor of mice for *in vivo* analysis and over the major clearance organs for *ex vivo* analysis. Background subtraction was performed using a mouse injected intravenously with PBS (100 µL PBS, 29G needle) and D-Luciferin delivered intraperitoneally as described above. Data were analyzed using the Living Image® software (PerkinElmer®).

Table S1: Thermocycler program for the amplification of the DNA template.

**Step**	**Cycle**	**Temperature**	**Time**
Denaturation	20 ×	98 °C	10 seconds
Annealing		64 °C	30 seconds
Extension		72 °C	30 seconds per kilobase
Final extension	1 ×	72 °C	2 minutes
Hold	1 ×	4 °C	Indefinite

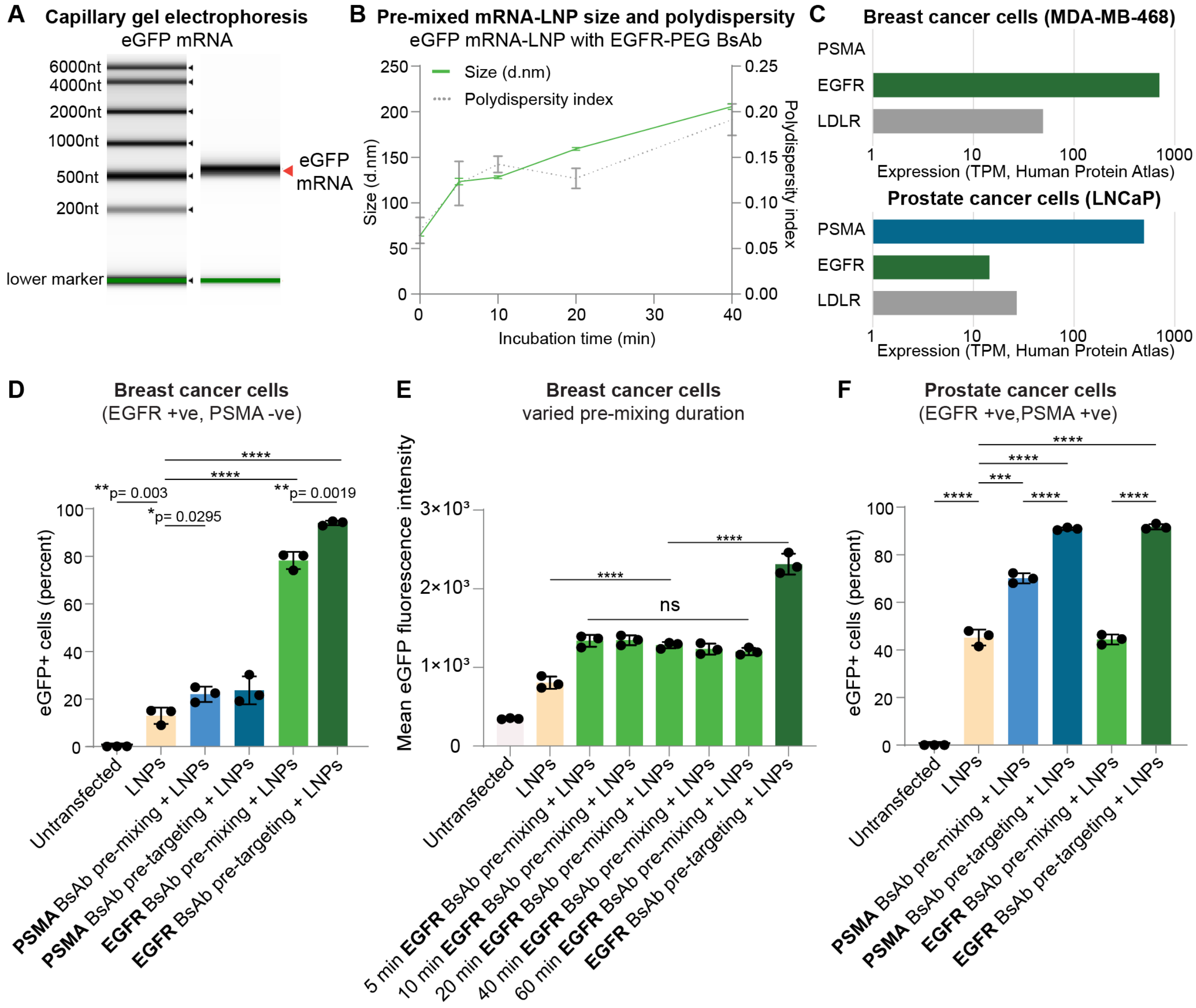

Figure S1: (A) Analysis of size and purity of *in vitro* transcribed eGFP mRNA on electropherogram. (B) Triplicate dynamic light scattering measurements of size and polydispersity index of eGFP-mRNA LNPs without BsAbs, and after different incubation times (pre-mixing) with EGFR-PEG BsAbs. (C) Quantification of PSMA, EGFR, and LDLR RNA in MDA-MB-468 breast cancer cells and LNCaP prostate cancer cells, respectively, in transcripts per million (TPM; Human Protein Atlas proteinatlas.org). (D) Percentage of eGFP expressing MDA-MB-468 breast cancer cells (EGFR+ve, PSMA-ve) transfected with eGFP-mRNA LNPs. EGFR-PEG BsAbs or PSMA-PEG BsAbs were pre-mixed with LNPs or pre-targeted to MDA-MB-468 cells, respectively. (E) Mean eGFP fluorescence intensity of MDA-MB-468 breast cancer cells transfected with eGFP-mRNA LNPs. EGFR-PEG BsAbs were pre-mixed with LNPs for different incubation times or pre-targeted to MDA-MB-468 cells, respectively. (F) Percentage of eGFP expressing LNCaP prostate cancer cells (EGFR+ve, PSMA+ve) transfected with eGFP-mRNA LNPs. EGFR-PEG BsAbs or PSMA-PEG BsAbs were pre-mixed with LNPs or pre-targeted to MDA-MB-468 cells, respectively. eGFP expression was measured using flow cytometry. Bars represent the mean value, error bars indicate standard deviation (n = 3). Statistical analysis using two-tailed t-tests assuming equal variance with ***p < 0.001 and ****p < 0.0001.

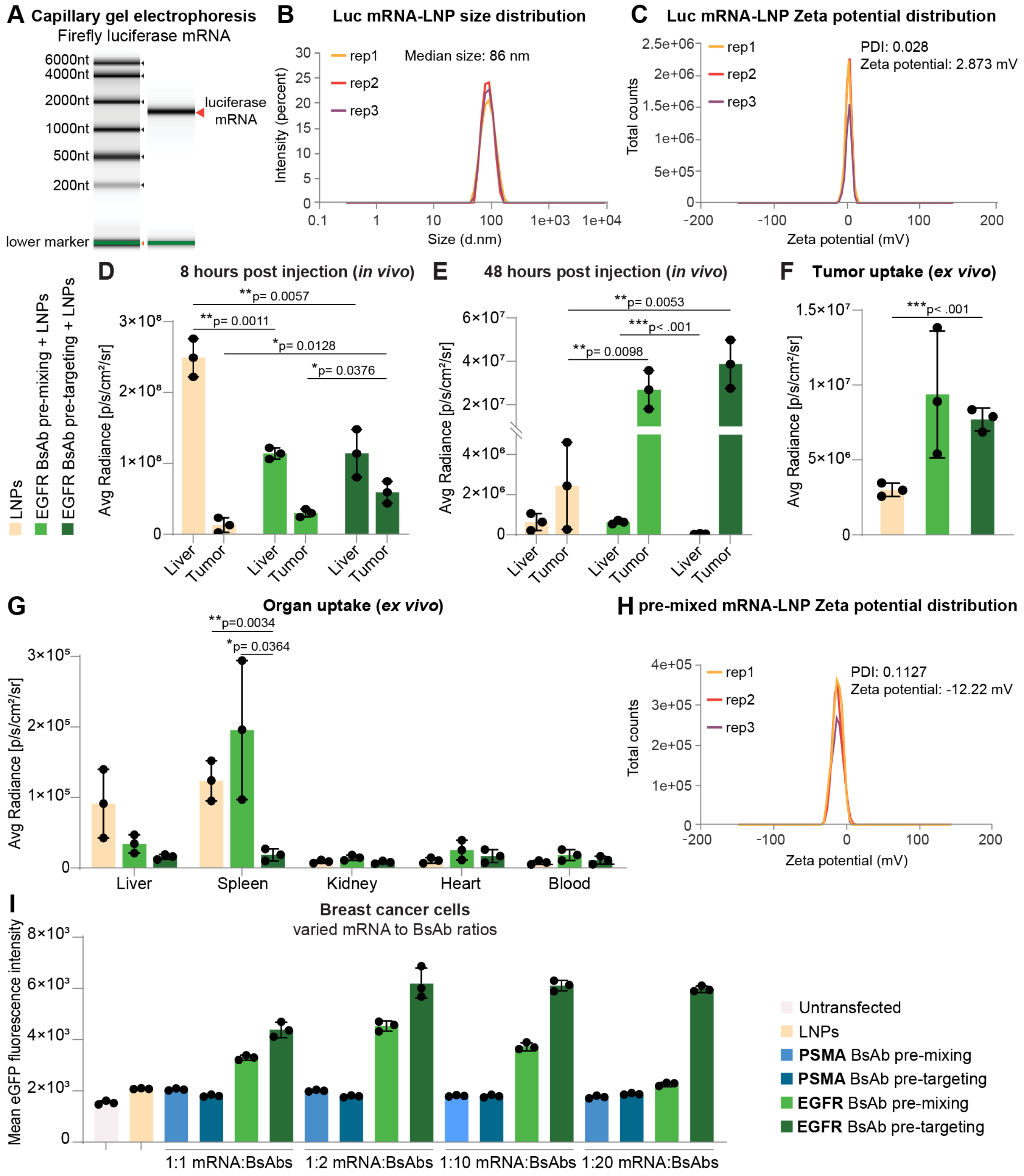

Figure S2: (A) Analysis of size and purity of *in vitro* transcribed firefly luciferase (Luc) mRNA on electropherogram. (B) Triplicate dynamic light scattering measurements of size distribution of luciferase-mRNA LNPs. (C) Triplicate electrophoretic light scattering measurements of zeta potential distribution of luciferase-mRNA LNPs. (D) *In vivo* average radiance measurement of bioluminescence in the liver compared to tumor for different targeting approaches eight hours and (E) 48 hours after luciferase mRNA-LNP administration. (F) *Ex vivo* average radiance measurement of bioluminescence in the tumor, (G) liver, spleen, kidney, heart, and blood. (H) Triplicate electrophoretic light scattering measurements of zeta potential distribution of eGFP-mRNA LNPs pre-mixed with PSMA BsAbs. (I) Mean eGFP fluorescence intensity of MDA-MB-468 breast cancer cells (PSMA-ve, EGFR+ve)

transfected with eGFP-mRNA LNPs. PSMA-PEG BsAbs or EGFR-PEG BsAbs were pre-mixed with LNPs or pre-targeted to MDA-MB-468 cells at varied mRNA to BsAb ratios, respectively. Mean eGFP fluorescence intensity was measured using flow cytometry. Background bioluminescence was subtracted based on a saline-injected mouse. Statistical analysis was performed using two-tailed t-tests assuming equal variance. Bars represent the mean value, error bars indicate standard deviation (n = 3).

Supplemental methods references

1. Henderson, J.M., Ujita, A., Hill, E., Yousif-Rosales, S., Smith, C., Ko, N., McReynolds, T., Cabral, C.R., Escamilla-Powers, J.R., and Houston, M.E. (2021). Cap 1 messenger RNA synthesis with co-transcriptional CleanCap(®) analog by in vitro transcription. Curr. Protoc. *1*, e39. 10.1002/cpz1.39.
2. Howard, C.B., Fletcher, N., Houston, Z.H., Fuchs, A.V., Boase, N.R., Simpson, J.D., Raftery, L.J., Ruder, T., Jones, M.L., de Bakker, C.J., Mahler, S.M., and Thurecht, K.J. (2016). Overcoming instability of antibody-nanomaterial conjugates: next generation targeted nanomedicines using bispecific antibodies. Adv. Healthc. Mater. *5*, 2055-2068. 10.1002/adhm.201600263.
3. Yang, X.D., Jia, X.C., Corvalan, J.R., Wang, P., and Davis, C.G. (2001). Development of ABX-EGF, a fully human anti-EGF receptor monoclonal antibody, for cancer therapy. Crit. Rev. Oncol. Hematol. *38*, 17-23. 10.1016/s1040-8428(00)00134-7.
4. Neil, B. (2006) Modified antibodies to prostate-specific membrane antigen and uses thereof. United States patent US20060088539, patent application 21956305.
5. FlowJo™ Software for Windows, Version 10.10.0 (2023). Becton, Dickinson and Company.
6. GraphPad Prism™ for Windows, Version 10.1.2. GraphPad Software, Boston, Massachusetts USA, www.graphpad.com.
7. Schindelin, J., Arganda-Carreras, I., Frise, E., Kaynig, V., Longair, M., Pietzsch, T., Preibisch, S., Rueden, C., Saalfeld, S., Schmid, B., et al. (2012). Fiji: an open-source platform for biological-image analysis. Nat. Methods *9*, 676-682. 10.1038/nmeth.2019.
8. Mastronarde, D.N. (2003). SerialEM: a program for automated tilt series acquisition on Tecnai microscopes using prediction of specimen position. Microsc. Microanal. *9*, 1182-1183. 10.1017/s1431927603445911.
9. Mastronarde, D.N. (2005). Automated electron microscope tomography using robust prediction of specimen movements. J. Struct. Biol. *152*, 36-51. 10.1016/j.jsb.2005.07.007.

10. Kremer, J.R., Mastronarde, D.N., and McIntosh, J.R. (1996). Computer visualization of three-dimensional image data using IMOD. J. Struct. Biol. *116*, 71-76. 10.1006/jsbi.1996.0013.
11. Mastronarde, D.N., and Held, S.R. (2017). Automated tilt series alignment and tomographic reconstruction in IMOD. J. Struct. Biol. *197*, 102-113. 10.1016/j.jsb.2016.07.011.
12. Fletcher, N.L., Prior, A., Choy, O., Humphries, J., Huda, P., Ghosh, S., Houston, Z.H., Bell, C.A., and Thurecht, K.J. (2022). Pre-targeting of polymeric nanomaterials to balance tumour accumulation and clearance. Chem. Commun. *58*, 7912-7915. 10.1039/D2CC02443H.
