# Supplementary material for: Targeted mRNA delivery with bispecific antibodies that tether LNPs to cell surface markers
Source: Mol Ther Nucleic Acids. 2025 Mar 19;36(2):102520. doi: 10.1016/j.omtn.2025.102520 (PMC11999258; doi:10.1016/j.omtn.2025.102520)
Supplement: Document S2. Article plus supplemental information [file mmc2.pdf]

Molecular Therapy
Nucleic Acids
Brief report

Targeted mRNA delivery with bispecific antibodies that tether LNPs to cell surface markers

Bettina Dietmair,[1,2,4] James Humphries,[1,3,4] Timothy R. Mercer,[1,2] Kristofer J. Thurecht,[1,3] Christopher B. Howard,[1] and Seth W. Cheetham[1,2]

[1]Australian Institute for Bioengineering and Nanotechnology, The University of Queensland, Brisbane, QLD, Australia; [2]BASE Facility, The University of Queensland, Brisbane, QLD, Australia; [3]Centre for Advanced Imaging, ARC Research Hub for Advanced Manufacture of Targeted Radiopharmaceuticals, The University of Queensland, St Lucia, QLD 4072, Australia

Efficient delivery of mRNA-lipid nanoparticles (LNPs) to specific cell types remains a major challenge for mRNA therapeutics. Conventional targeting approaches involve modifying the lipid composition or functionalizing the surface of LNPs, which complicates manufacturing and alters nanoparticle size, charge, and stealth, impacting their delivery and immunogenicity. Here, we present a generalizable method for targeted mRNA-LNP delivery that uses bispecific antibodies (BsAbs) to form a bridge between LNPs and cell surface markers. BsAbs can be combined with LNPs or administered first, binding to surface proteins on target cells and later retaining unmodified LNPs in affected tissues. We demonstrate the efficient and cell-type-specific delivery of mRNA-LNPs beyond the liver, targeting epidermal growth factor receptor (EGFR)- and folate hydrolase 1 (PSMA)-positive cells *in vitro* and *in vivo*. The flexibility of this technology, achieved by substituting the cell-targeting region of the BsAbs, enables the rapid development of next-generation targeted mRNA drugs.

INTRODUCTION

mRNA therapies are rapidly emerging as a new class of drugs with the potential to treat a wide range of human diseases. Beyond vaccines, mRNA drugs in development include treatments for cancers,[1,2] autoimmunity,[3] and hereditary diseases.[4] The efficacy of mRNA drugs depends on the ability to deliver mRNA efficiently to specific cell types. Polyethylene glycol (PEG)-coated lipid nanoparticles (LNPs) are clinically validated as safe and effective mRNA delivery systems.[5–7] However, following intravenous administration, LNP accumulation and mRNA expression occur mainly in the liver.[8,9] The liver tropism of LNPs limits the potential of mRNA for treating extrahepatic diseases.

Current attempts to target mRNA-LNPs *in vivo* employ high drug doses and remain restricted to the liver, lungs, and spleen.[10,11] We report a customizable solution to achieve cell-specific mRNA delivery to previously inaccessible tissues using bispecific antibodies (BsAbs). The BsAbs comprise two linked single-chain variable fragments (scFvs) that can bind to PEG on the exterior of LNPs and to a protein enriched on the target cell surface. BsAbs can be attached to the surface of LNPs before administration (pre-mixing). We further present pre-targeting, a novel approach for targeted mRNA-LNP delivery, whereby cells are initially exposed to BsAbs, followed by administration of unmodified LNPs.[12] Targeting with BsAbs enabled efficient mRNA drug delivery beyond the liver. Sequential administration of BsAbs and mRNA-LNPs demonstrated specificity for epidermal growth factor receptor (EGFR) and folate hydrolase 1 (PSMA) *in vitro* and *in vivo*, significantly improving mRNA-LNP delivery to the target tissue and reducing accumulation in off-target organs. The new concept of pre-targeting can be adapted to different cell surface antigens to achieve safe and efficacious targeted mRNA delivery, enabling the development of mRNA medicines for broader applications.

RESULTS

Pre-targeting with BsAbs improves cell-specific mRNA-LNP delivery

To determine if cell surface antigen-specific BsAbs enable targeted mRNA-LNP delivery, we synthesized and encapsulated enhanced green fluorescent protein (EGFP) mRNA in LNPs (Figure S1A). As active targeting approaches commonly attach the targeting agent to the LNPs (pre-mixing; Figure 1A), not to the target cells (pre-targeting; Figure 1B), we first characterized the effect of surface functionalization on LNP properties. Pre-mixing of the mRNA-LNPs with BsAbs increased the median size of the mRNA-LNP from 82 to 181 and 412 nm for anti-PSMA and anti-EGFR BsAbs, respectively (Figure 1C). LNP size and polydispersity doubled after 5 min of incubation with anti-EGFR BsAbs and tripled by 40 min (Figure S1B). BsAb

Received 10 December 2024; accepted 16 March 2025;
https://doi.org/10.1016/j.omtn.2025.102520.

[4]These authors contributed equally

Correspondence: Christopher B. Howard, Australian Institute for Bioengineering and Nanotechnology, The University of Queensland, Brisbane, QLD, Australia.
E-mail: c.howard2@uq.edu.au
Correspondence: Seth W. Cheetham, Australian Institute for Bioengineering and Nanotechnology, The University of Queensland, Brisbane, QLD, Australia.
E-mail: s.cheetham@uq.edu.au

Published by Elsevier Inc. on behalf of The American Society of Gene and Cell Therapy.
This is an open access article under the CC BY license (http://creativecommons.org/licenses/by/4.0/).

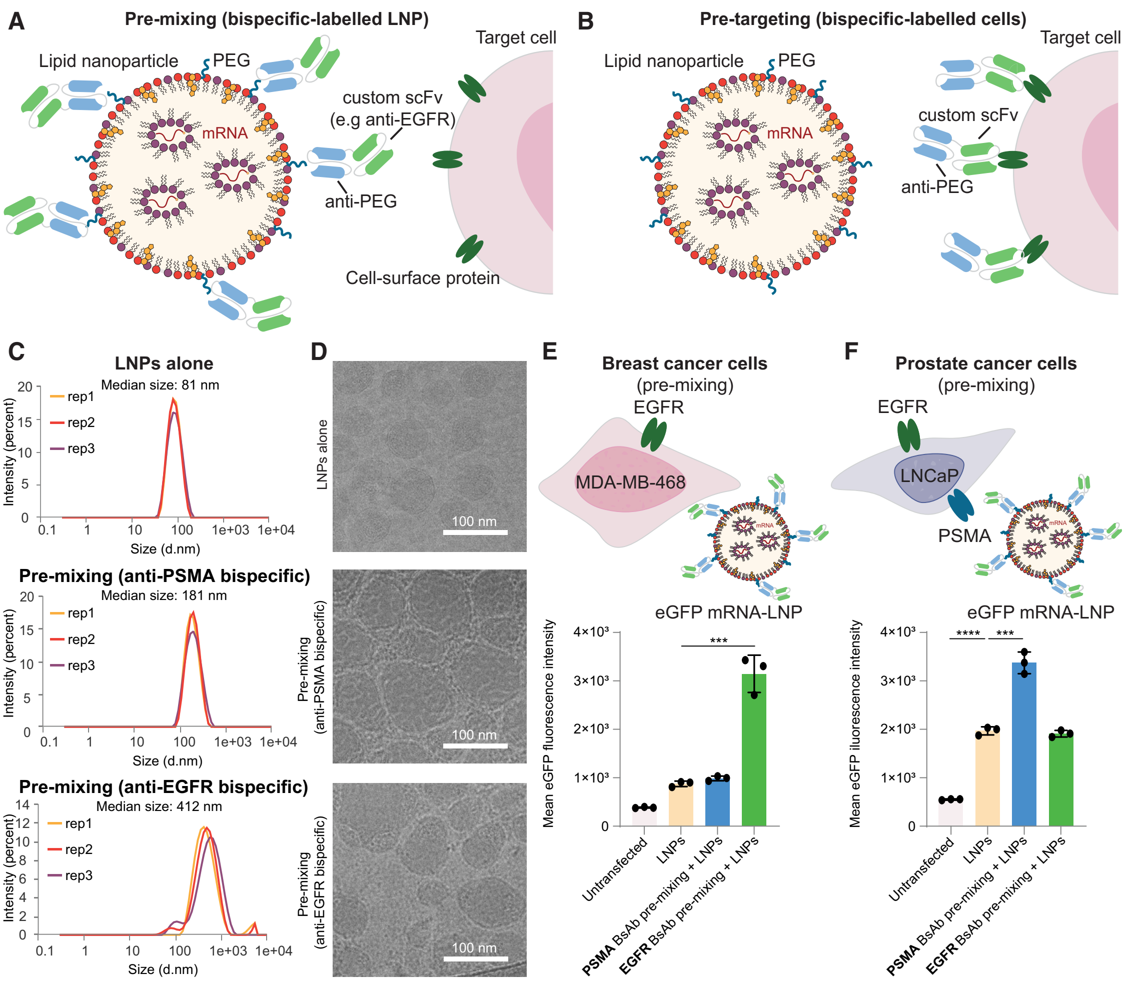

Figure 1. Pre-mixing with bispecific antibodies alters physicochemical properties and delivery of mRNA-LNPs

(A) During pre-mixing, bispecific antibodies (BsAbs) bind to polyethylene glycol (PEG) on the surface of mRNA-loaded lipid nanoparticles (LNPs). The second binding region of the BsAb can bind to the surface protein on the target cell. (B) For pre-targeting, cells are exposed to BsAbs that can specifically bind to surface proteins. mRNA-carrying LNPs can then bind to the PEG-specific binding region of the BsAb. (C) Triplicate dynamic light scattering measurements of size distribution of EGFP-mRNA LNPs without BsAbs, after pre-mixing with PSMA-PEG BsAbs, and after pre-mixing with EGFR-PEG BsAbs. (D) Cryogenic transmission electron microscopy images of EGFP-mRNA LNPs without BsAbs, after pre-mixing with PSMA-PEG BsAbs, and after pre-mixing with EGFR-PEG BsAbs. Scale bar: 100 nm. (E and F) Mean EGFP fluorescence intensity of cells transfected with untargeted EGFP-mRNA LNPs, with LNPs pre-mixed with PSMA-PEG BsAbs, or LNPs pre-mixed with EGFR-PEG BsAbs, respectively, for (E) MDA-MB-468 breast cancer cells (PSMA−ve and EGFR+ve) and (F) LNCaP prostate cancer cells (PSMA+ve and EGFR+ve). Mean EGFP fluorescence intensity was measured using flow cytometry. Statistical analysis was performed using two-tailed t tests assuming equal variance. Bars represent the mean value, and error bars indicate standard deviation ($n = 3$). ***$p < 0.001$ and ****$p < 0.0001$.

coating altered LNP morphology, reduced particle uniformity, and induced aggregation (Figures 1C and 1D), which may contribute to LNP behavior *in vivo*.[13,14] For intravenous administration and tissue penetration, nanoparticle sizes below 150 nm are preferred.[15]

To evaluate cell-type-specific delivery of mRNA-LNPs pre-mixed with BsAbs, we used MDA-MB-468 breast cancer cells, which express EGFR but not PSMA, and LNCaP prostate cancer cells, which express both EGFR and PSMA (Figure S1C). Both cell lines express the low-density lipoprotein receptor (LDLR), and cell culture medium was supplemented with serum containing apolipoprotein,[16] as the uptake of untargeted LNPs *in vivo* is largely dependent on apolipoprotein E adsorption to the nanoparticle surface and subsequent internalization via the LDLR on hepatocytes.[17] Pre-mixing of LNPs with anti-PEG:anti-EGFR BsAbs enhanced mRNA-LNP delivery to MDA-MB-468 (Figure 1E). LNP surface functionalization with anti-PEG:anti-PSMA BsAbs did not improve mRNA-LNP delivery to the PSMA-negative cell line. To confirm the cell-type specificity of BsAb-mediated targeting, we analyzed mRNA-LNP delivery to EGFR+ve (EGFR-positive), PSMA+ve (PSMA-positive) LNCaP prostate cancer cells (Figure 1F). EGFP mRNA-LNP delivery significantly improved when mRNA-LNPs were pre-mixed with anti-PEG:anti-PSMA BsAbs. Pre-mixing with

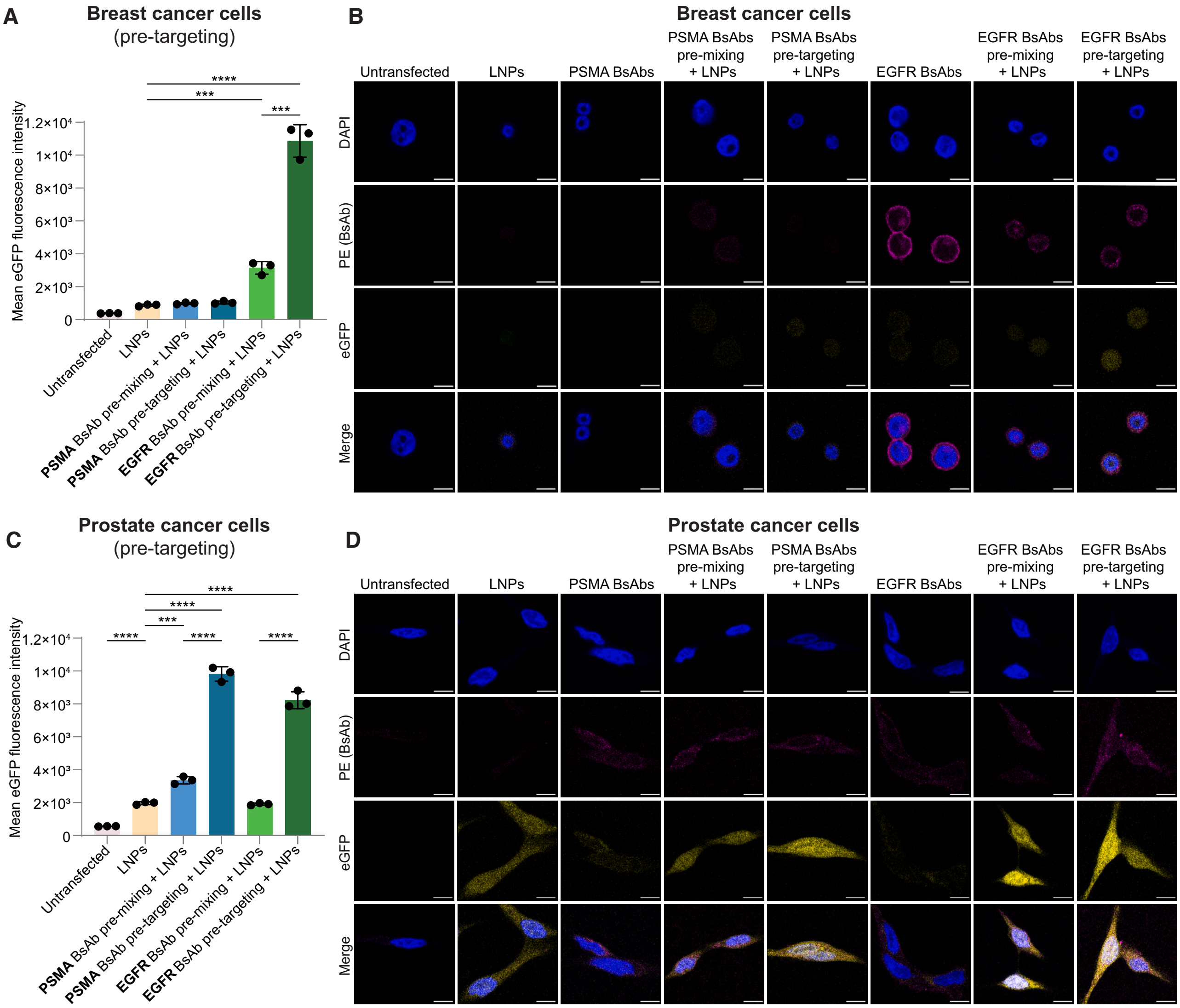

Figure 2. Pre-targeting of cells with bispecific antibodies improves cell-specific delivery of mRNA-LNPs

(A) Mean EGFP fluorescence intensity of MDA-MB-468 breast cancer cells (PSMA−ve and EGFR+ve) transfected with EGFP-mRNA LNPs. PSMA-PEG BsAbs or EGFR-PEG BsAbs were pre-mixed with LNPs or pre-targeted to MDA-MB-468 cells, respectively. (B) Confocal microscopy images of MDA-MB-468 after addition of EGFP mRNA-LNPs, PSMA-PEG, or EGFR-PEG BsAbs, BsAbs pre-mixed with LNPs, or pre-targeting with BsAbs followed by addition of LNPs. (C) Mean EGFP fluorescence intensity of LNCaP prostate cancer cells (PSMA+ve and EGFR+ve) transfected with EGFP-mRNA LNPs. PSMA-PEG BsAbs or EGFR-PEG BsAbs were pre-mixed with LNPs or pre-targeted to LNCaP cells, respectively. (D) Confocal microscopy images of LNCaP after addition of EGFP mRNA-LNPs, PSMA-PEG, or EGFR-PEG BsAbs, BsAbs pre-mixed with LNPs, or pre-targeting with BsAbs followed by addition of LNPs. Confocal microscopy images were taken at 63× magnification and show EGFP expression (yellow), BsAb localization (protein L-phycoerythrin conjugate labeled; magenta), and 4′,6-diamidino-2-phenylindole DNA stain (DAPI; blue) 4 h after addition of LNPs. Scale bar: 10 μm. Mean EGFP fluorescence intensity was measured using flow cytometry. Statistical analysis was performed using two-tailed t tests assuming equal variance. Bars represent the mean value, and error bars indicate standard deviation ($n = 3$). ***$p < 0.001$ and ****$p < 0.0001$.

anti-PEG:anti-EGFR BsAbs had no effect on EGFP expression compared to untargeted LNPs.

We then compared the established pre-mixing protocol to the pre-targeting of cells with BsAbs. Pre-targeting MDA-MB-468 breast cancer cells with anti-PEG:anti-EGFR BsAbs significantly increased EGFP expression compared to pre-mixing (12- vs. 3-fold improvement over mRNA-LNPs alone; Figures 2A, 2B, and S1D). The duration of pre-mixing had no impact on mRNA-LNP delivery (Figure S1E). Similarly, pre-targeting LNCaP prostate cancer cells reached almost 3-fold improvement with PSMA BsAbs and 4-fold improvement with EGFR BsAbs compared to pre-mixing (Figures 2C, 2D, and S1F). Compared to untargeted LNP transfection, pre-targeting with EGFR-PEG and PSMA-PEG achieved 4- and

5-fold EGFP expression, respectively. Notably, pre-targeting with EGFR-PEG BsAbs enhanced mRNA-LNP delivery to EGFR+ve LNCaP cells, while pre-mixing did not improve uptake.

In summary, pre-mixing and pre-targeting with anti-PEG BsAbs facilitate cell-type-specific mRNA-LNP delivery *in vitro*. The pre-targeting approach is applicable to different cell lines and target antigens and is more efficient than pre-mixing. Active targeting employs interactions with a cell surface receptor to promote specificity and internalization via endocytosis.[18] As BsAbs are below the size range in which receptor-mediated endocytosis is triggered,[19,20] they accumulate on the plasma membrane and are internalized once mRNA-LNPs bind (Figures 2B and 2D). In addition to maintaining the physicochemical properties of mRNA-LNPs by pre-targeting, avidity might improve delivery with more BsAb binding sites available on cell surfaces during pre-targeting than PEG on LNPs during pre-mixing. Increased avidity could explain improved mRNA-LNP delivery to LNCaP cells when pre-targeting with EGFR-PEG BsAbs despite lower EGFR expression on the cell surface (Figure S1C). As pre-targeting MDA-MB-468 with EGFR-PEG BsAbs achieved the greatest improvement over untargeted and pre-mixed LNP delivery *in vitro*, we tested this condition *in vivo*.

Pre-targeting with BsAbs improves targeted mRNA-LNP delivery *in vivo*

To evaluate the efficacy of BsAb-targeted mRNA-LNP delivery *in vivo*, we synthesized and encapsulated firefly luciferase mRNA in LNPs (Figures S2A–S2C) and pre-mixed or pre-targeted with EGFR-PEG BsAbs for intravenous administration to BALB/c nude mice with subcutaneous MDA-MB-468 xenografts (Figure 3A). Pre-mixing and pre-targeting with EGFR-PEG BsAbs significantly increased mRNA-LNP delivery to the tumor tissue (over 8- and 7-fold) while reducing the radiance in the liver by a third and half compared to untargeted LNPs, respectively, 8 h after luciferase mRNA-LNP administration (Figures 3B and S2D). After 48 h, luciferase expression in the liver was reduced across all delivery groups (Figures 3C and S2E). In contrast, luminescence in the tumor remained consistent with the 8-h time point in pre-targeted animals, maintaining significant levels over untargeted LNPs and only decreasing by around 10%. Radiance in the pre-mixing group tumors was reduced by 60% compared to the measurement after 8 h.

Ex vivo analysis of tumor signal confirmed *in vivo* biodistribution results, showing comparable luminescence between pre-mixing and pre-targeting cohorts with more than 4.6- and 3.5-fold elevated radiance compared to untargeted mRNA-LNP administration, respectively (Figures 3D and S2F). Consistently, total flux in the liver was highest in the untargeted LNP group and significantly lower in mice treated with pre-targeting compared to pre-mixing of BsAbs and LNPs (Figures 3E and S2G). Likewise, spleen uptake was the lowest in pre-targeted mice compared to over 5- and 8-fold increased spleen signals for the administration of untargeted and pre-mixed mRNA-LNPs, respectively (Figure 3F). The high splenic uptake of LNPs pre-mixed with BsAbs could result from changes in physicochemical properties, including a large protein corona around the LNPs[14,21] and a charge shift from positively charged, unmodified LNPs to negatively charged, BsAb pre-mixed LNPs[22] (Figure S2H). Lower spleen accumulation of unmodified mRNA LNPs in the pre-targeting group, compared to untargeted LNPs, could be a result of higher tumor uptake, leaving less nanoparticles in circulation for off-target accumulation. Radiance was low in the heart, blood, and the second major clearance organ, kidney (Figures 3G and S2G).

Confirming the *in vitro* results, targeting mRNA-LNPs with EGFR-PEG BsAbs *in vivo* resulted in mRNA delivery to EGFR+ve tumors and reduced delivery to the liver, demonstrating specificity and efficacy compared to delivery of untargeted LNPs. Pre-targeting led to sustained mRNA expression in the tumor and the lowest signals in the liver and spleen compared to pre-mixing of LNPs with BsAbs or administration of untargeted LNPs.

DISCUSSION

We demonstrated efficient mRNA-LNP targeting *in vitro* and *in vivo* using BsAbs specific to LNPs and cell surface markers. Temporal separation of BsAb and nanoparticle administration via pre-targeting achieved superior mRNA uptake and expression. This is the first time pre-targeting has been characterized for mRNA-LNPs. One explanation for the improved biodistribution of mRNA-LNPs during pre-targeting, relative to pre-mixed systems, could be reduced protein fouling on the particle surface.[12] Reduced protein adsorption to the LNP surface can decrease receptor-mediated hepatic uptake[17] and immune recognition,[23] thus extending the circulation time and enabling the nanoparticles to reach peripheral target tissues. Maintained particle properties, including size and charge, can facilitate LNP uptake into target cells. Pre-targeting further conserves ligand affinity *in vivo*, which can vary in conjugated antibody systems due to reaction conditions and the attachment of antibodies in an unfavorable orientation.[24] BsAbs enable mRNA delivery to previously inaccessible tissues, which broadens the scope of conditions addressable with mRNA therapeutics. Lower mRNA dosage and reduced uptake in off-target organs, such as the liver and spleen, could reduce the toxicity of mRNA therapies. While mRNA-LNPs were cleared from the metabolically active liver,[23] efficient mRNA expression persisted exclusively in the tumor. The structure of the BsAbs facilitates substitution of the antigen-specific binding domain,[25–27] enabling customized targeting of unmodified, PEG-containing mRNA carriers to markers present on individual patient cell types. The separate production of the mRNA-LNP and targeting agent (BsAb) greatly streamlines manufacture and quality control compared to functionalized mRNA-LNPs.[28] Broad applicability to PEGylated nanocarriers and decreased complexity of production and purification enhance the scalability of pre-targeting.[29] The well-established clinical use and safety of mRNA-LNPs[30] and BsAbs[31] provide strong precedence for a streamlined clinical translation of this technology. While sequential drug administration is common in cancer therapy,[32] this treatment regimen should be considered for clinical practice in other applications. Efficient and adaptable targeting of

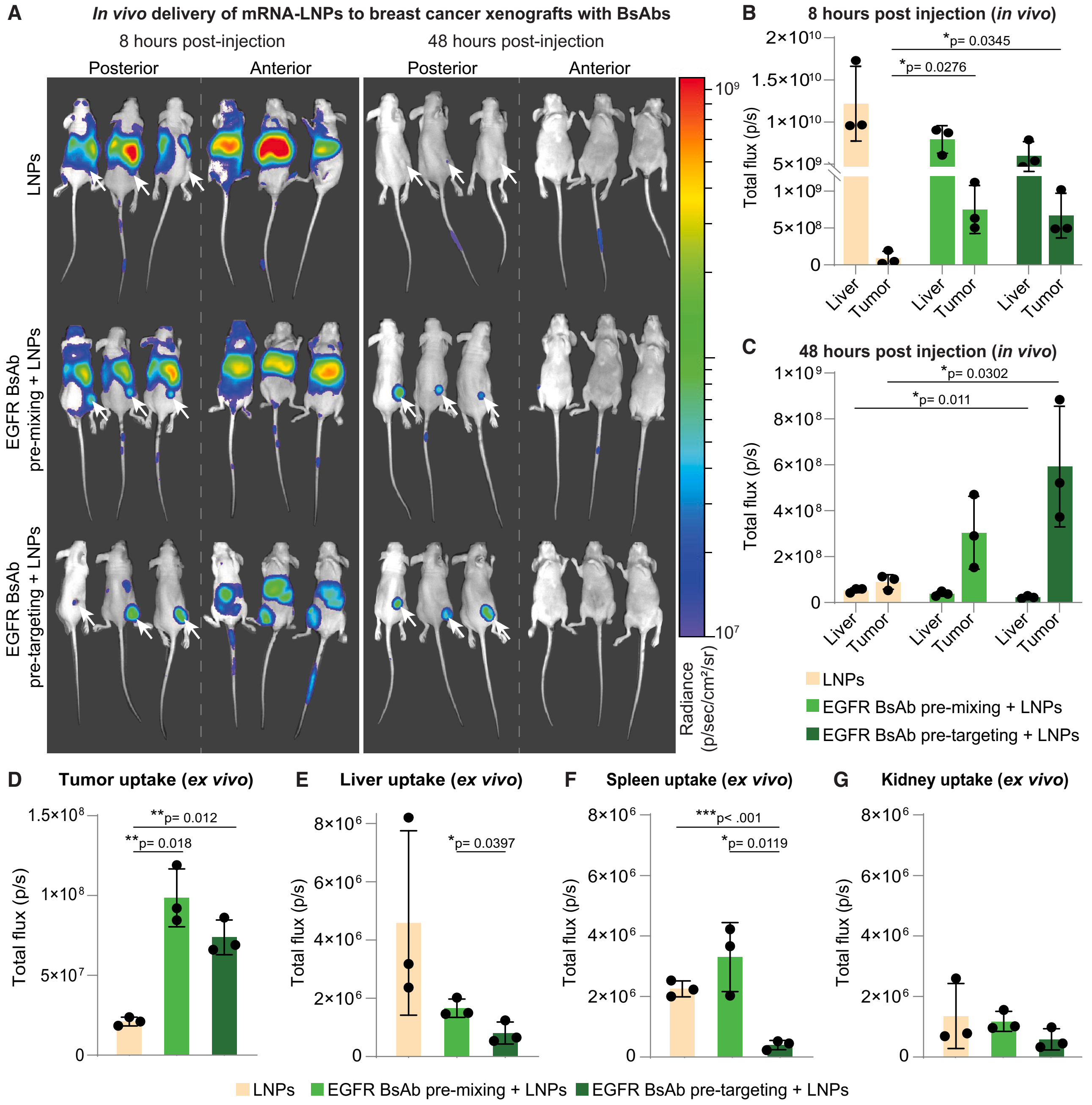

Figure 3. Pre-targeting with bispecific antibodies improves targeted delivery of mRNA-LNPs *in vivo*

(A) *In vivo* bioluminescence images of MDA-MB-468 tumor-bearing mice injected with untargeted luciferase mRNA-LNPs, LNPs pre-mixed with EGFR-PEG BsAbs, or mice pre-injected with EGFR-PEG BsAbs followed by administration of mRNA-LNPs. White arrows indicate posterior tumor localization. (B and C) *In vivo* bioluminescence in the liver compared to tumor for different targeting approaches (B) 8 and (C) 48 h after luciferase mRNA-LNP administration. (D–G) *Ex vivo* bioluminescence imaging of (D) tumor, (E) liver, (F) spleen, and (G) kidney tissue at 48 h post-injection. Background was subtracted based on a saline-injected mouse. Statistical analysis was performed using two-tailed t tests assuming equal variance. Bars represent the mean value, and error bars indicate standard deviation (n = 3).

mRNA-LNPs enables the rapid development of next-generation mRNA drugs, including protein replacement therapies and gene editing applications for incurable diseases.

MATERIALS AND METHODS

Detailed methods can be found in the supplemental materials and methods.

DATA AVAILABILITY

Data supporting the findings of this study are available within the article and its supplemental information.

ACKNOWLEDGMENTS

We acknowledge the following sources of funding and support: the Australian Government Research Training Program (RTP) Scholarship to B.D., Innovation Connections funding to S.W.C. and T.R.M., the National Health and Medical Research Council (GNT2019056 to K.J.T. and GNT2014002 and GNT1161832 to T.R.M.), the Australian Research Council (IH220100017 to K.J.T. and DE230100036 to S.W.C.), the Medical Research Future Fund (MRFCRI000063 and MRFNCRI000089 to S.W.C. and T.R.M.), the National Collaborative Research Infrastructure Strategy (NCRIS) to T.R.M. and S.W.C., Therapeutic Innovation Australia (TIA) to T.R.M. and S.W.C., Tour de Cure to S.W.C., and The University of Queensland to S.W.C. and T.R.M. The authors acknowledge the facilities and the scientific and technical assistance of the Australian National Fabrication Facility (ANFF, Queensland Node), the Centre for Microscopy and Microanalysis (CMM), the National Imaging Facility (NIF), and BASE at The University of Queensland. BASE is supported by TIA. TIA is supported by the Australian government through the National Collaborative Research Infrastructure Strategy (NCRIS) program.

AUTHOR CONTRIBUTIONS

B.D. and J.H. performed the experiments and the analysis. C.B.H. and S.W.C. conceived the project. S.W.C., C.B.H., T.R.M., and K.J.T. funded the study. All authors contributed to writing the paper.

DECLARATION OF INTERESTS

T.R.M. and S.W.C. have received research funding from Oxford Nanopore Technologies, Sartorius Stedim Australia, and Sanofi. T.R.M. and S.W.C. have received support for conference attendance, travel, and accommodations from Moderna and Oxford Nanopore Technologies.

SUPPLEMENTAL INFORMATION

Supplemental information can be found online at https://doi.org/10.1016/j.omtn.2025.102520.

REFERENCES

1. Lopez, J., Powles, T., Braiteh, F., Siu, L.L., LoRusso, P., Friedman, C.F., Balmanoukian, A.S., Gordon, M., Yachnin, J., Rottey, S., et al. (2025). Autogene cevumeran with or without atezolizumab in advanced solid tumors: a phase 1 trial. Nat. Med. *31*, 152–164. https://doi.org/10.1038/s41591-024-03334-7.

2. Lam, P.Y., Omer, N., Wong, J.K.M., Tu, C., Alim, L., Rossi, G.R., Victorova, M., Tompkins, H., Lin, C.Y., Mehdi, A.M., et al. (2025). Enhancement of anti-sarcoma immunity by NK cells engineered with mRNA for expression of a EphA2-targeted CAR. Clin. Transl. Med. *15*, e70140. https://doi.org/10.1002/ctm2.70140.

3. Liu, Y., Zhang, R., Qiu, N., Wang, S., Chen, J., Xu, X., Xiang, J., and Shen, Y. (2024). Spleen-targeted mRNA nanoparticles for modulating B cell hyperactivation in rheumatoid arthritis therapy. Adv. Funct. Mater. *35*, 2417101. https://doi.org/10.1002/adfm.202417101.

4. Koeberl, D., Schulze, A., Sondheimer, N., Lipshutz, G.S., Geberhiwot, T., Li, L., Saini, R., Luo, J., Sikirica, V., Jin, L., et al. (2024). Interim analyses of a first-in-human phase 1/2 mRNA trial for propionic acidaemia. Nature *628*, 872–877. https://doi.org/10.1038/s41586-024-07266-7.

5. Adams, D., Gonzalez-Duarte, A., O'Riordan, W.D., Yang, C.C., Ueda, M., Kristen, A.V., Tournev, I., Schmidt, H.H., Coelho, T., Berk, J.L., et al. (2018). Patisiran, an RNAi therapeutic, for hereditary transthyretin amyloidosis. N. Engl. J. Med. *379*, 11–21. https://doi.org/10.1056/NEJMoa1716153.

6. Polack, F.P., Thomas, S.J., Kitchin, N., Absalon, J., Gurtman, A., Lockhart, S., Perez, J.L., Pérez Marc, G., Moreira, E.D., Zerbini, C., et al. (2020). Safety and efficacy of the BNT162b2 mRNA Covid-19 vaccine. N. Engl. J. Med. *383*, 2603–2615. https://doi.org/10.1056/NEJMoa2034577.

7. Baden, L.R., El Sahly, H.M., Essink, B., Kotloff, K., Frey, S., Novak, R., Diemert, D., Spector, S.A., Rouphael, N., Creech, C.B., et al. (2021). Efficacy and safety of the mRNA-1273 SARS-CoV-2 vaccine. N. Engl. J. Med. *384*, 403–416. https://doi.org/10.1056/NEJMoa2035389.

8. Rizvi, F., Everton, E., Smith, A.R., Liu, H., Osota, E., Beattie, M., Tam, Y., Pardi, N., Weissman, D., and Gouon-Evans, V. (2021). Murine liver repair via transient activation of regenerative pathways in hepatocytes using lipid nanoparticle-complexed nucleoside-modified mRNA. Nat. Commun. *12*, 613. https://doi.org/10.1038/s41467-021-20903-3.

9. Song, D., Zhao, Y., Wang, Z., and Xu, Q. (2024). Tuning lipid nanoparticles for RNA delivery to extrahepatic organs. Adv. Mater. *36*, 2401445. https://doi.org/10.1002/adma.202401445.

10. Su, K., Shi, L., Sheng, T., Yan, X., Lin, L., Meng, C., Wu, S., Chen, Y., Zhang, Y., Wang, C., et al. (2024). Reformulating lipid nanoparticles for organ-targeted mRNA accumulation and translation. Nat. Commun. *15*, 5659. https://doi.org/10.1038/s41467-024-50093-7.

11. Cheng, Q., Wei, T., Farbiak, L., Johnson, L.T., Dilliard, S.A., and Siegwart, D.J. (2020). Selective organ targeting (SORT) nanoparticles for tissue-specific mRNA delivery and CRISPR–Cas gene editing. Nat. Nanotechnol. *15*, 313–320. https://doi.org/10.1038/s41565-020-0669-6.

12. Fletcher, N.L., Prior, A., Choy, O., Humphries, J., Huda, P., Ghosh, S., Houston, Z.H., Bell, C.A., and Thurecht, K.J. (2022). Pre-targeting of polymeric nanomaterials to balance tumour accumulation and clearance. Chem. Commun. *58*, 7912–7915. https://doi.org/10.1039/D2CC02443H.

13. Daniel, S., Kis, Z., Kontoravdi, C., and Shah, N. (2022). Quality by design for enabling RNA platform production processes. Trends Biotechnol. *40*, 1213–1228. https://doi.org/10.1016/j.tibtech.2022.03.012.

14. Lam, K., Schreiner, P., Leung, A., Stainton, P., Reid, S., Yaworski, E., Lutwyche, P., and Heyes, J. (2023). Optimizing lipid nanoparticles for delivery in primates. Adv. Mater. *35*, 2211420. https://doi.org/10.1002/adma.202211420.

15. Dave, V., Tak, K., Sohgaura, A., Gupta, A., Sadhu, V., and Reddy, K.R. (2019). Lipid-polymer hybrid nanoparticles: Synthesis strategies and biomedical applications. J. Microbiol. Methods *160*, 130–142. https://doi.org/10.1016/j.mimet.2019.03.017.

16. Lee, D.Y., Lee, S.Y., Yun, S.H., Jeong, J.W., Kim, J.H., Kim, H.W., Choi, J.S., Kim, G.-D., Joo, S.T., Choi, I., and Hur, S.J. (2022). Review of the current research on fetal bovine serum and the development of cultured meat. Food Sci. Anim. Resour. *42*, 775–799. https://doi.org/10.5851/kosfa.2022.e46.

17. Akinc, A., Maier, M.A., Manoharan, M., Fitzgerald, K., Jayaraman, M., Barros, S., Ansell, S., Du, X., Hope, M.J., Madden, T.D., et al. (2019). The Onpattro story and the clinical translation of nanomedicines containing nucleic acid-based drugs. Nat. Nanotechnol. *14*, 1084–1087. https://doi.org/10.1038/s41565-019-0591-y.

18. Vetter, V.C., and Wagner, E. (2022). Targeting nucleic acid-based therapeutics to tumors: Challenges and strategies for polyplexes. J. Contr. Release *346*, 110–135. https://doi.org/10.1016/j.jconrel.2022.04.013.

19. O'Connor, I.B., and Wall, J.G. (2018). 16 - Immobilization of antibodies on cardiovascular stents. In Functionalised Cardiovascular Stents, J.G. Wall, H. Podbielska, and M. Wawrzyńska, eds. (Woodhead Publishing), pp. 319–341. https://doi.org/10.1016/B978-0-08-100496-8.00017-2.

20. Zhang, S., Li, J., Lykotrafitis, G., Bao, G., and Suresh, S. (2009). Size-dependent endocytosis of nanoparticles. Adv. Mater. *21*, 419–424. https://doi.org/10.1002/adma.200801393.

21. Cataldi, M., Vigliotti, C., Mosca, T., Cammarota, M., and Capone, D. (2017). Emerging role of the spleen in the pharmacokinetics of monoclonal antibodies, nanoparticles and exosomes. Int. J. Mol. Sci. *18*, 1249. https://doi.org/10.3390/ijms18061249.

22. Amruta, A., Iannotta, D., Cheetham, S.W., Lammers, T., and Wolfram, J. (2023). Vasculature organotropism in drug delivery. Adv. Drug Deliv. Rev. *201*, 115054. https://doi.org/10.1016/j.addr.2023.115054.

23. Saber, N., Senti, M.E., and Schiffelers, R.M. (2024). Lipid nanoparticles for nucleic acid delivery beyond the liver. Hum. Gene Ther. *35*, 617–627. https://doi.org/10.1089/hum.2024.106.

24. Menon, I., Zaroudi, M., Zhang, Y., Aisenbrey, E., and Hui, L. (2022). Fabrication of active targeting lipid nanoparticles: Challenges and perspectives. Mater. Today Adv. *16*, 100299. https://doi.org/10.1016/j.mtadv.2022.100299.

25. Janowicz, P.W., Houston, Z.H., Bunt, J., Fletcher, N.L., Bell, C.A., Cowin, G., Howard, C.B., Taslima, D., Westra van Holthe, N., Prior, A., et al. (2022). Understanding nanomedicine treatment in an aggressive spontaneous brain cancer model at the stage of early blood brain barrier disruption. Biomaterials *283*, 121416. https://doi.org/10.1016/j.biomaterials.2022.121416.

26. Gao, Z., Li, X., Zhao, K., Geng, H., Zhang, P., Ju, Y., Huda, P., Howard, C.B., Thurecht, K.J., Ashokkumar, M., et al. (2022). Confined microemulsion sono-polymerization of poly(ethylene glycol) nanoparticles for targeted delivery. Chem. Commun. *58*, 7777–7780. https://doi.org/10.1039/D2CC01874H.

27. Moles, E., Howard, C.B., Huda, P., Karsa, M., McCalmont, H., Kimpton, K., Duly, A., Chen, Y., Huang, Y., Tursky, M.L., et al. (2023). Delivery of PEGylated liposomal doxorubicin by bispecific antibodies improves treatment in models of high-risk childhood leukemia. Sci. Transl. Med. *15*, eabm1262. https://doi.org/10.1126/scitranslmed.abm1262.

28. Parhiz, H., Shuvaev, V.V., Pardi, N., Khoshnejad, M., Kiseleva, R.Y., Brenner, J.S., Uhler, T., Tuyishime, S., Mui, B.L., Tam, Y.K., et al. (2018). PECAM-1 directed re-targeting of exogenous mRNA providing two orders of magnitude enhancement of vascular delivery and expression in lungs independent of apolipoprotein E-mediated uptake. J. Contr. Release *291*, 106–115. https://doi.org/10.1016/j.jconrel.2018.10.015.

29. Su, Y.C., Burnouf, P.A., Chuang, K.H., Chen, B.M., Cheng, T.L., and Roffler, S.R. (2017). Conditional internalization of PEGylated nanomedicines by PEG engagers for triple negative breast cancer therapy. Nat. Commun. *8*, 15507. https://doi.org/10.1038/ncomms15507.

30. Vlatkovic, I. (2021). Non-immunotherapy application of LNP-mRNA: maximizing efficacy and safety. Biomedicines 9, 530.

31. Nejadghaderi, S.A., Balibegloo, M., Noori, M., Fayyaz, F., Saghazadeh, A., and Rezaei, N. (2023). Clinical efficacy and safety of bispecific antibodies for the treatment of solid tumors: a systematic review and meta-analysis. Expert Rev. Anticancer Ther. *23*, 307–318. https://doi.org/10.1080/14737140.2023.2183847.

32. Wu, M., Huang, X., Chen, M., and Zhang, Y. (2024). Administration sequences in single-day chemotherapy regimens for breast cancer: a comprehensive review from a practical perspective. Front. Oncol. *14*, 1353067. https://doi.org/10.3389/fonc.2024.1353067.

OMTN, Volume *36*

Supplemental information

Targeted mRNA delivery with bispecific antibodies that tether LNPs to cell surface markers

Bettina Dietmair, James Humphries, Timothy R. Mercer, Kristofer J. Thurecht, Christopher B. Howard, and Seth W. Cheetham

Supplemental materials and methods

Template production

eGFP and firefly luciferase mRNA templates were designed with the CleanCap® AG promoter,[1] human alpha-globin 5′ UTR and the mouse alpha-globin 3′ UTR sequences and synthesized as gBlocks™ HiFi Gene Fragments (IDT™). 0.5 ng of Gene Fragments, forward primer (10 µM), reverse primer (10 µM), and NEB® Q5® HotStart 2 × master mix were combined at room temperature in a 100 µL PCR reaction and placed on ice. The template was amplified using the thermocycler program described in **Table S1**. Eight PCR reactions were purified using the Qiagen® QIAquick® PCR cleanup kit, according to the manufacturer's instructions. The PCR product was eluted in 30 µL of ultrapure water, analyzed by gel electrophoresis and quantified using UV spectrophotometry.

mRNA production

Amplified Gene Fragments were used as template for mRNA *in vitro* transcription (IVT) at a concentration of 50 µg/mL. IVT was performed using 16 µg/mL T7 RNA polymerase (New England Biolabs; NEB® M0251), ribonucleotides (6 mM ATP, 5 mM CTP, 5 mM GTP; NEB®), 5 mM N1-methylpseudouridine-5'-triphosphate (TriLink® BioTechnologies, TRN1081), 4 mM CleanCap® AG reagent (TriLink® BioTechnologies, TRN7113), transcription buffer (40 mM Tris·HCl pH 8.0, 16.5 mM magnesium acetate, 10 mM dithiothreitol (DTT), 20 mM spermidine, 0.002 % (v/v) Triton X-100), 2 U/mL yeast inorganic pyrophosphatase (NEB®) and 1000 U/mL murine RNase inhibitor (NEB®). The IVT reaction was incubated at 37 °C for three hours and terminated by incubation with 200 U/mL NEB® DNase I at 37 °C for 15 minutes. The mRNA product was purified using a Monarch® RNA Cleanup Kit (NEB®) according to the manufacturer's instructions, eluted in 1 mM sodium citrate and sterile filtered with a 0.22 µm syringe filter. The mRNA was quantified by UV spectrophotometry and integrity confirmed using the Agilent® TapeStation™.

LNP production

For LNP formulation, a total lipid concentration of 15 mg/mL was used in the molar ratio of 50 SM-102 : 10 DSPC : 38.5 cholesterol : 1.5 DMG-PEG2000. The lipid mixture was made up to 3.75 mL with molecular grade 100 % ethanol. 1.5 mg of purified mRNA were combined with 11.25 mL of 0.1 M sodium acetate, pH 4.0. Formulation was performed on the NanoAssemblr® Ignite™ platform with the following parameters: total volume 13.5 mL, total flow rate 12 mL/min and flow rate ratio (aqueous:organic) 3:1. The mRNA-LNPs were dialyzed using the Slide-A-Lyzer™ dialysis cassette

(10K MWCO) and concentrated using an Amicon® Ultra-15 Centrifugal Filter Unit (10 kDa MWCO). The concentrated mRNA-LNPs were filtered with a 0.22 µm syringe filter and 0.2 volumes of 50 % sucrose were added by gentle pipetting for a final 10 % concentration. Encapsulation efficiency was measured on an Infinite® 200 PRO microplate reader (Tecan®) using the Quant-iT™ RiboGreen® RNA Reagent Kit (Life Technologies™). Determined encapsulation efficiencies for eGFP and luciferase mRNA-LNPs were 98.0 % and 98.8 %, respectively.

Bispecific antibody production

Bispecific antibodies were produced as previously described.[2] An scFv specific for PEG was linked to an scFv specific for human epidermal growth factor receptor[3] or folate hydrolase 1[4] via a glycine serine linker (G4S). The BsAb sequences were codon optimized for expression in *C. griseus* cells, included a κ light chain leader sequence for protein secretion, a 6 × Histidine motif at the N-terminus of the BsAb and a c-myc epitope tag at the C-terminus for purification and detection of the BsAb. The BsAb genes were cloned into the pcDNA™ 3.1 (+) mammalian expression plasmid (Invitrogen™) using HindIII and NotI restriction sites. For transient transfection, the plasmid DNA was transfected into ExpiCHO™ cells (Gibco™) using 2 µg DNA per mL cells at a concentration of 6 × 10^6 mL^{-1} cells. For a 200 mL cell volume transfection, 200 µg DNA in 8 mL of OptiPRO™ serum free medium (SFM; Gibco™) were mixed with 7.4 mL OptiPRO™ SFM containing 640 µL ExpiFectamine™ (Gibco™) for five minutes prior to transfecting ExpiCHO™ cells. The transfected cells were cultured in ExpiCHO™ expression medium (Gibco™) at 37 °C, 7.5% CO_2, 70 % humidity, with shaking at 130 rpm for 24 h, before feeding with 10 % ExpiCHO™ Feed (Gibco™) and 1.2 mL ExpiFectamine™ enhancer reagent (Gibco™) and returning cultures to 32 °C, 7.5 % CO_2, 70 % humidity, with shaking at 130 rpm.

Following transfection, the cells were pelleted by centrifugation at 5250 × g for 30 minutes and the supernatant was collected and filtered through a 0.22 µm PES membrane (Sartorius®). The BsAbs were purified from the supernatant using a 5 mL HisTrap™ excel column (Cytiva™), eluting the protein with 20 × 10^{-3} M sodium phosphate, 500 × 10^{-3} M sodium chloride, and 500 × 10^{-3} M Imidazole pH 7.4. BsAbs were then buffer exchanged into 1 × phosphate-buffered saline (PBS) using a HiPrep™ 26/10 column (Cytiva™). The final product was sterile filtered using a 0.2 µm polyethersulfone (PES) membrane filter (Sartorius®).

Characterisation of LNPs

LNP size and charge were measured on a Zetasizer® Ultra (Malvern Panalytical®). mRNA-LNPs were diluted 50-fold in distilled water (Invitrogen™). For pre-mixing, 10 × excess BsAbs (w/w) were added and samples were incubated for 60 minutes at room temperature. Ten-fold BsAb excess was used as lower ratios did not achieve maximum efficacy and higher ratios reduced delivery using the pre-mixing method (**Figure S2I**).

Cell culture

MDA-MB-468 human breast cancer cells were cultured in Dulbecco's modified Eagle's medium (DMEM; Gibco™), supplemented with 10 % (v/v) fetal bovine serum (FBS; Gibco™) and 1 × penicillin-streptomycin (P/S; 100 U/mL penicillin and 100 µg/mL streptomycin; Gibco™). LNCaP human prostate cancer cells were maintained in Roswell Park Memorial Institute medium (RPMI-1640; Sigma-Aldrich®), 10 % FBS and 1 × P/S. Cells were incubated at 37 °C in 5 % CO_2 and propagated for no more than 30 passages.

***In vitro* BsAb-targeted mRNA-LNP delivery**

For 70 % confluency at transfection, 1.9×10^5 MDA-MB-468 or LNCaP cells were plated into the wells of 24-well cell culture plates in 500 µL of appropriate cell culture medium. To enhance LNCaP adhesion, 24-well plates were coated with poly-D-lysine (Gibco™) according to manufacturer specifications. The cells were allowed to attach overnight before treatment. All treatments were incubated with cells for 60 minutes at 37 °C with 5 % CO_2. Treatments were diluted in Dulbecco's Phosphate-Buffered Saline (DPBS; Gibco™) and added in volumes of 10 µL. mRNA concentration per well was 60 ng and BsAb concentration was 600 ng. For washing steps, cells were rinsed twice with DPBS and fresh complete medium was added. Four hours after addition of LNPs, cells were prepared for flow cytometry.

For pre-mixed samples, eGFP mRNA-LNPs were incubated with BsAbs for 60 minutes at room temperature. The treatment was then added to the wells, incubated and washed. For pre-targeting, cells were incubated with BsAbs for 60 minutes, washed, incubated with eGFP mRNA-LNPs and washed again. The untargeted LNP samples were incubated with DPBS, washed, incubated with eGFP mRNA-LNPs and washed again.

Flow cytometry

To prepare samples for flow cytometry, medium was removed, cells were washed with DPBS, detached with 0.25 % 1 × trypsin (Gibco™), centrifuged at 200 × g for 5 minutes and resuspended in 250 µL flow buffer (DPBS, 2 % FBS, 2 mM ethylenediaminetetraacetic acid; EDTA). 7-aminoactinomycin D (7-AAD; Invitrogen™) viability stain was used and samples were incubated in the dark on ice for 30 minutes. 20,000 single cell events were recorded on a CytoFLEX™ Flow Cytometer (Beckman Coulter®) at a flow rate of 10 µL/s. eGFP and 7-AAD were excited using a 488 nm laser and emission was detected with a 525/40 bandpass and a 690/50 bandpass filter, respectively. Compensation and data analysis were performed using FlowJo™ v10.10.0. Software[5] (BD Life Sciences™). GraphPad Prism™ v10.1.2[6] (GraphPad Software) was used for statistical analysis and graphing. Statistical analysis was performed using two-tailed t-tests assuming equal variance with *$p < 0.05$, **$p < 0.01$, ***$p < 0.001$ and ****$p < 0.0001$.

Confocal microscopy

4×10^4 MDA-MB-468 cells or 6×10^4 LNCaP cells were seeded on coverslips in 24-well plates and incubated overnight at 37 °C with 5 % CO_2. For LNCaP cells, coverslips were coated with poly-D-lysine (Gibco™) according to manufacturer instructions. The cells were treated as described for *in vitro* BsAb-targeted mRNA-LNP delivery. To prepare the samples for confocal microscopy, the cells were fixed with 4 % paraformaldehyde (Novachem™) for 15 minutes at room temperature, washed three times with 1 × PBS (Gibco™), permeabilized and blocked with PBS buffer containing 0.1 % Triton X-100 (Sigma-Aldrich®) and 1 × bovine serum albumin (Sigma-Aldrich®) for one hour at room temperature. DAPI (BioLegend®) and Protein L-phycoerythrin conjugate staining (1:100; Cell Signaling Technology®) was performed for one hour at room temperature. The cells were washed three times with 1 × PBS and one time with Milli-Q® water before mounting on glass slides. Imaging was performed on a Zeiss® LSM® 710 inverted laser scanning confocal microscope at the Queensland node of the NCRIS-enabled Australian National Fabrication Facility (ANFF). Images were taken using a 63 × oil immersion objective for sequential scanning with excitation of DAPI, eGFP and phycoerythrin at 405 nm, 488 nm and 561 nm, respectively. FIJI[7] was used for image processing.

Cryo electron microscopy

Cryo-transmission electron microscopy was performed by the Centre for Microscopy and Microanalysis, UQ. Lacey carbon films on 300 mesh copper grids were used as sample carriers (Electron Microscopy

Sciences®) and glow discharged for 60 seconds at 10 % power using a Femto system for sample cleaning directly before usage (Diener Electronic®). The samples were cryo-fixed in liquid ethane at -184 ºC using a Leica EM-GP2 (Leica® Microsystems). 2.5 µL samples were blotted for three to four seconds, frozen at 20 ºC and 99 % relative humidity, and stored in liquid nitrogen until further processing. Image acquisition of the frozen hydrated LNPs was performed using a CryoARM™ 200 JEM-Z200 FSC (JEOL™) equipped with a K2™ Summit direct electron detector (Gatan®). The microscope was operated at 200 kV with the microscope energy filter set to a slit width of 20 eV. SerialEM[8,9] in combination with Gatan's Digital Micrograph was used for image acquisition. Samples were imaged at -180 ºC under low dose conditions. The exposure time for each record was 4 seconds with 40 subframes in counting mode. The dose rate on the detector was 8.5 e/px/s. Movie frames were motion corrected with the program's frame alignment feature and saved as a sum image of 40 subframes.[10,11]

***In vivo* imaging of mRNA-LNP biodistribution**

All studies were in accordance with guidelines of the Animal Ethics Committee of The University of Queensland (UQ; Approval 2023/AE000135), and the Australian Code for the Care and Use of Animals for Scientific Purposes. Female Balb/c nude mice (approximately 8 weeks of age) were acquired from the Ozgene-ARC® (Western Australia) and housed in temperature and humidity-controlled housing with *ad libitum* access to food and water.

Mice were subcutaneously injected with 5×10^6 MDA-MB-468 cells in the right flank (50 µL PBS, 27G needle). After tumors reached a palpable size (ca. 100-200 mm^3), the mice were segregated into three cohorts of n = 3 animals where the targeting strategy was varied. Mice in the untargeted LNP group were injected intravenously via the lateral tail vein with 10 µg of luciferase mRNA-LNPs diluted in 100 µL of PBS (29G needle). Mice in the pre-mixed cohort were injected intravenously with mRNA-LNPs pre-incubated with anti-PEG:anti-EGFR BsAbs at a ratio of 50 µg BsAbs per 10 µg mRNA 15 minutes prior to intravenous injection (diluted in 100 µL PBS, 29G needle). Mice in the pre-targeted cohort were injected intravenously with 1 mg of anti-PEG:anti-EGFR BsAbs (diluted in 100 µL of PBS with 300 mM NaCl) eight hours[12] prior to injection of 10 µg of mRNA-LNPs diluted in 100 µL of PBS (29G needle).

Mice were injected intraperitoneally with 150 mg kg^{-1} of D-Luciferin (VivoGlo™) 15 minutes prior to imaging using an IVIS® Lumina™ X5 imaging system (PerkinElmer®) eight hours and 48 hours post

injection of the LNPs. Data were acquired using the default acquisition settings, then post-processed to a binning factor of four. Segmented regions of interest (ROIs) were drawn to delineate the liver and tumor of mice for *in vivo* analysis and over the major clearance organs for *ex vivo* analysis. Background subtraction was performed using a mouse injected intravenously with PBS (100 µL PBS, 29G needle) and D-Luciferin delivered intraperitoneally as described above. Data were analyzed using the Living Image® software (PerkinElmer®).

Table S1: Thermocycler program for the amplification of the DNA template.

**Step**	**Cycle**	**Temperature**	**Time**
Denaturation	20 ×	98 °C	10 seconds
Annealing		64 °C	30 seconds
Extension		72 °C	30 seconds per kilobase
Final extension	1 ×	72 °C	2 minutes
Hold	1 ×	4 °C	Indefinite

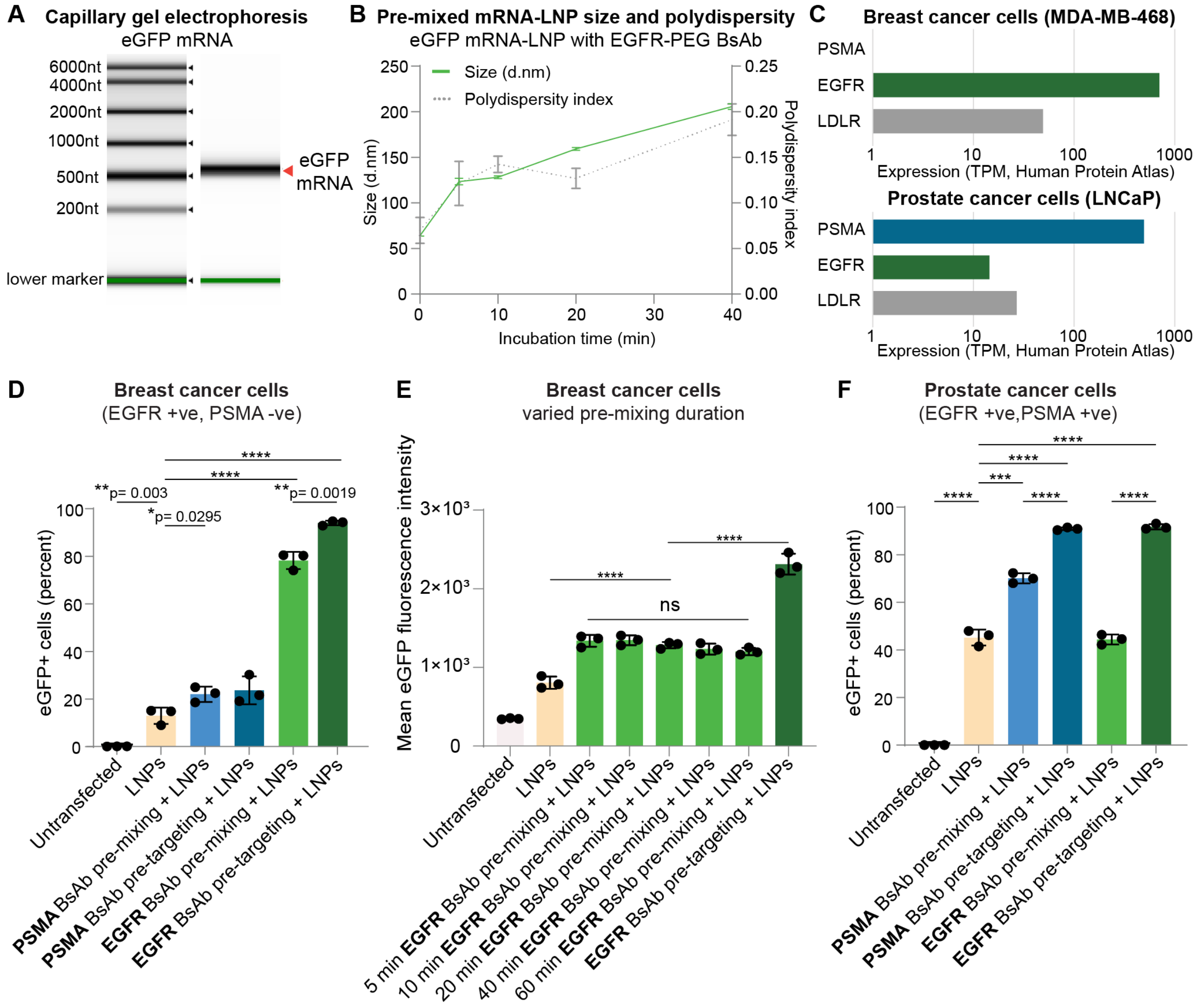

Figure S1: (A) Analysis of size and purity of *in vitro* transcribed eGFP mRNA on electropherogram. (B) Triplicate dynamic light scattering measurements of size and polydispersity index of eGFP-mRNA LNPs without BsAbs, and after different incubation times (pre-mixing) with EGFR-PEG BsAbs. (C) Quantification of PSMA, EGFR, and LDLR RNA in MDA-MB-468 breast cancer cells and LNCaP prostate cancer cells, respectively, in transcripts per million (TPM; Human Protein Atlas proteinatlas.org). (D) Percentage of eGFP expressing MDA-MB-468 breast cancer cells (EGFR+ve, PSMA-ve) transfected with eGFP-mRNA LNPs. EGFR-PEG BsAbs or PSMA-PEG BsAbs were pre-mixed with LNPs or pre-targeted to MDA-MB-468 cells, respectively. (E) Mean eGFP fluorescence intensity of MDA-MB-468 breast cancer cells transfected with eGFP-mRNA LNPs. EGFR-PEG BsAbs were pre-mixed with LNPs for different incubation times or pre-targeted to MDA-MB-468 cells, respectively. (F) Percentage of eGFP expressing LNCaP prostate cancer cells (EGFR+ve, PSMA+ve) transfected with eGFP-mRNA LNPs. EGFR-PEG BsAbs or PSMA-PEG BsAbs were pre-mixed with LNPs or pre-targeted to MDA-MB-468 cells, respectively. eGFP expression was measured using flow cytometry. Bars represent the mean value, error bars indicate standard deviation (n = 3). Statistical analysis using two-tailed t-tests assuming equal variance with ***$p < 0.001$ and ****$p < 0.0001$.

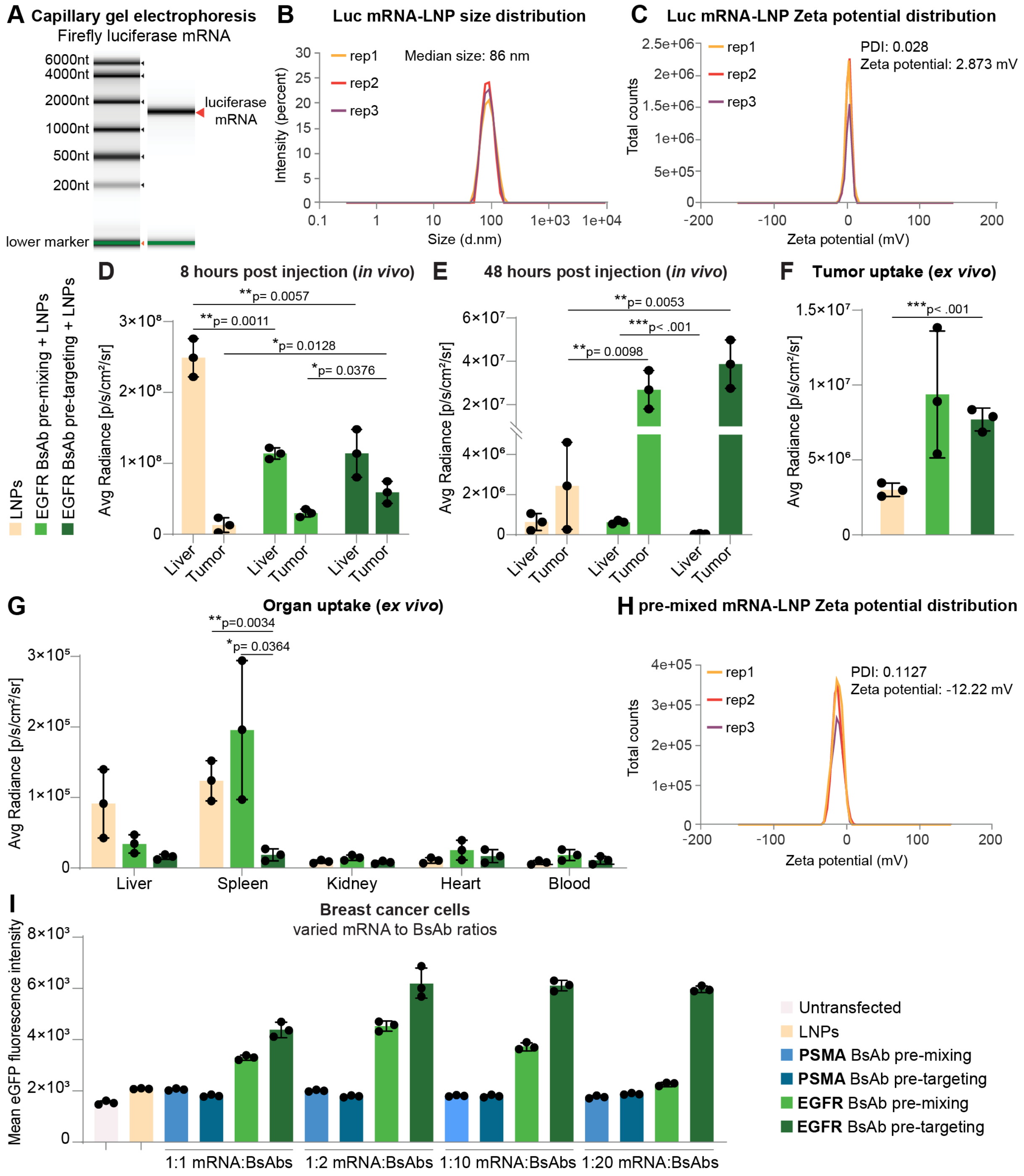

Figure S2: (A) Analysis of size and purity of *in vitro* transcribed firefly luciferase (Luc) mRNA on electropherogram. (B) Triplicate dynamic light scattering measurements of size distribution of luciferase-mRNA LNPs. (C) Triplicate electrophoretic light scattering measurements of zeta potential distribution of luciferase-mRNA LNPs. (D) *In vivo* average radiance measurement of bioluminescence in the liver compared to tumor for different targeting approaches eight hours and (E) 48 hours after luciferase mRNA-LNP administration. (F) *Ex vivo* average radiance measurement of bioluminescence in the tumor, (G) liver, spleen, kidney, heart, and blood. (H) Triplicate electrophoretic light scattering measurements of zeta potential distribution of eGFP-mRNA LNPs pre-mixed with PSMA BsAbs. (I) Mean eGFP fluorescence intensity of MDA-MB-468 breast cancer cells (PSMA-ve, EGFR+ve)

transfected with eGFP-mRNA LNPs. PSMA-PEG BsAbs or EGFR-PEG BsAbs were pre-mixed with LNPs or pre-targeted to MDA-MB-468 cells at varied mRNA to BsAb ratios, respectively. Mean eGFP fluorescence intensity was measured using flow cytometry. Background bioluminescence was subtracted based on a saline-injected mouse. Statistical analysis was performed using two-tailed t-tests assuming equal variance. Bars represent the mean value, error bars indicate standard deviation (n = 3).

Supplemental methods references

1. Henderson, J.M., Ujita, A., Hill, E., Yousif-Rosales, S., Smith, C., Ko, N., McReynolds, T., Cabral, C.R., Escamilla-Powers, J.R., and Houston, M.E. (2021). Cap 1 messenger RNA synthesis with co-transcriptional CleanCap(®) analog by in vitro transcription. Curr. Protoc. *1*, e39. 10.1002/cpz1.39.
2. Howard, C.B., Fletcher, N., Houston, Z.H., Fuchs, A.V., Boase, N.R., Simpson, J.D., Raftery, L.J., Ruder, T., Jones, M.L., de Bakker, C.J., Mahler, S.M., and Thurecht, K.J. (2016). Overcoming instability of antibody-nanomaterial conjugates: next generation targeted nanomedicines using bispecific antibodies. Adv. Healthc. Mater. *5*, 2055-2068. 10.1002/adhm.201600263.
3. Yang, X.D., Jia, X.C., Corvalan, J.R., Wang, P., and Davis, C.G. (2001). Development of ABX-EGF, a fully human anti-EGF receptor monoclonal antibody, for cancer therapy. Crit. Rev. Oncol. Hematol. *38*, 17-23. 10.1016/s1040-8428(00)00134-7.
4. Neil, B. (2006) Modified antibodies to prostate-specific membrane antigen and uses thereof. United States patent US20060088539, patent application 21956305.
5. FlowJo™ Software for Windows, Version 10.10.0 (2023). Becton, Dickinson and Company.
6. GraphPad Prism™ for Windows, Version 10.1.2. GraphPad Software, Boston, Massachusetts USA, www.graphpad.com.
7. Schindelin, J., Arganda-Carreras, I., Frise, E., Kaynig, V., Longair, M., Pietzsch, T., Preibisch, S., Rueden, C., Saalfeld, S., Schmid, B., et al. (2012). Fiji: an open-source platform for biological-image analysis. Nat. Methods *9*, 676-682. 10.1038/nmeth.2019.
8. Mastronarde, D.N. (2003). SerialEM: a program for automated tilt series acquisition on Tecnai microscopes using prediction of specimen position. Microsc. Microanal. *9*, 1182-1183. 10.1017/s1431927603445911.
9. Mastronarde, D.N. (2005). Automated electron microscope tomography using robust prediction of specimen movements. J. Struct. Biol. *152*, 36-51. 10.1016/j.jsb.2005.07.007.

10. Kremer, J.R., Mastronarde, D.N., and McIntosh, J.R. (1996). Computer visualization of three-dimensional image data using IMOD. J. Struct. Biol. *116*, 71-76. 10.1006/jsbi.1996.0013.
11. Mastronarde, D.N., and Held, S.R. (2017). Automated tilt series alignment and tomographic reconstruction in IMOD. J. Struct. Biol. *197*, 102-113. 10.1016/j.jsb.2016.07.011.
12. Fletcher, N.L., Prior, A., Choy, O., Humphries, J., Huda, P., Ghosh, S., Houston, Z.H., Bell, C.A., and Thurecht, K.J. (2022). Pre-targeting of polymeric nanomaterials to balance tumour accumulation and clearance. Chem. Commun. *58*, 7912-7915. 10.1039/D2CC02443H.
